# Novel Marine Secondary Metabolites Worthy of Development as Anticancer Agents: A Review

**DOI:** 10.3390/molecules26195769

**Published:** 2021-09-23

**Authors:** Florence Nwakaego Mbaoji, Justus Amuche Nweze, Liyan Yang, Yangbin Huang, Shushi Huang, Akachukwu Marytheresa Onwuka, Ikechukwu Emmanuel Peter, Cynthia Chioma Mbaoji, Mingguo Jiang, Yunkai Zhang, Lixia Pan, Dengfeng Yang

**Affiliations:** 1 Guangxi Key Laboratory of Marine Natural Products and Combinatorial Biosynthesis Chemistry, Guangxi Beibu Gulf Marine Research Center, Guangxi Academy of Sciences, Nanning 530007, China; florence.mbaoji@unn.edu.ng (F.N.M.); justus.nweze@unn.edu.ng (J.A.N.); yangbinhuang106@163.com (Y.H.); hshushi@gxas.cn (S.H.); 2 College of Life Science and Technology of Guangxi University, Nanning 530004, China; 3 Department of Pharmacology and Toxicology, Faculty of Pharmaceutical Sciences, University of Nigeria, Nsukka 410001, Enugu State, Nigeria; akachukwu.onwuka@unn.edu.ng (A.M.O.); ikechukwu.peter@unn.edu.ng (I.E.P.); cynthia.mbaoji.194479@unn.edu.ng (C.C.M.); 4 Department of Science Laboratory Technology, Faculty of Physical Sciences, University of Nigeria, Nsukka 410001, Enugu State, Nigeria; 5 Department of Ecosystem Biology, Faculty of Science, University of South Bohemia in Ceske Budejovice, 37005 Ceske Budejovice, Czech Republic; 6 Soil and Water Research Infrastructure, Biology Centre, Czech Academy of Sciences, 37005 Ceske Budejovice, Czech Republic; 7 Guangxi Biomass Industrialization Engineering Institute, National Engineering Research Center of Non-Food Biorefinery, State Key Laboratory of Non-Food Biomass, Guangxi Academy of Sciences, Nanning 530007, China; yangliyan.1988@163.com; 8 Guangxi Key Laboratory for Polysaccharide Materials and Modifications, School of Marine Sciences and Biotechnology, Guangxi University for Nationalities, Nanning 530008, China; mzxyjiang@163.com

**Keywords:** cancer, marine secondary metabolites, lead optimization, cancer cell line, drug development

## Abstract

Secondary metabolites from marine sources have a wide range of biological activity. Marine natural products are promising candidates for lead pharmacological compounds to treat diseases that plague humans, including cancer. Cancer is a life-threatening disorder that has been difficult to overcome. It is a long-term illness that affects both young and old people. In recent years, significant attempts have been made to identify new anticancer drugs, as the existing drugs have been useless due to resistance of the malignant cells. Natural products derived from marine sources have been tested for their anticancer activity using a variety of cancer cell lines derived from humans and other sources, some of which have already been approved for clinical use, while some others are still being tested. These compounds can assault cancer cells via a variety of mechanisms, but certain cancer cells are resistant to them. As a result, the goal of this review was to look into the anticancer potential of marine natural products or their derivatives that were isolated from January 2019 to March 2020, in cancer cell lines, with a focus on the class and type of isolated compounds, source and location of isolation, cancer cell line type, and potency (IC_50_ values) of the isolated compounds that could be a guide for drug development.

## 1. Introduction

For decades, marine natural products have been sources of bioactive compounds with remarkable biological activities. These include anticancer, antimicrobial, antidiabetic, anti-inflammatory, antiparasitic, antihypertensive, and antioxidant activities. They can be traced to the effects of numerous marine compounds from different classes of constituents such as alkaloids, terpenoids, steroids, flavonoids, peptides, proteins, polyphenols, carotenoids, vitamins, tocopherols, tocotrienols, peptides, carbohydrates, etc. Not only marine compounds, but also terrestrial plant constituents have anticancer properties [1,2,3,4,5], as well as a plethora of other biological activities. The activities of these compounds have been screened in various disease conditions including cancer using in vivo or in vitro methods, and the documented evidence of these activities is reviewed regularly [6,7,8,9,10]. In vitro or cell-based screening assays, according to the National Cancer Institute (NCI), are used to screen and identify test compounds that have a direct cytotoxic effect or have an effect on cell proliferation, eventually leading to cell death [11]. 

Compounds with an outstanding activity profile in terms of efficacy and potency are chosen for further optimization and subsequent development into new drugs. Screening compounds for anticancer activity is time consuming. 

However, in accordance with the with the NCI’s disease-oriented drug screening approach for anticancer agents, the testing of compounds for anticancer activity involves the use of cancer cell lines or xenografts to achieve the desired effect. There has recently been a paradigm shift away from cytotoxic chemotherapy and towards target drug discovery. Target drug discovery entails the identification of molecular targets and the development of drugs that target those targets by the application of high-throughput molecular screening of hits. The selected hits are then subjected to biological activity confirmation in a living cell, followed by a determination of the new compound’s specificity using adequate cancer cell lines containing target receptors [12]. 

Cancer is synonymous with a neoplasm or malignant tumor. It is a deadly disease that lacks growth control and has the ability to invade neighboring tissues and organs of the body from its origin and eventually destroys them. Cancer is a genetic disease caused by alterations to genes that control the way cells function, grow, and divide. According to the National Cancer Institute (NCI), genetic changes can cause cancer. Cancer-causing genetic changes can be inherited from parents or develop during a person’s lifetime as a result of cell division errors or DNA damage caused by exposures to certain environmental factors [13]. Cancer-causing environmental factors include arsenic, benzene, and butane found in tobacco smoke, as well as ultraviolet rays from the sun [13]. Cancer is the world’s second leading cause of death after cardiovascular disease and was responsible for the death of 9.6 million people in 2018 [14].

Tumors are classified as benign or malignant. Benign tumors are non-aggressive in nature because they are localized to a particular site in the body and can surgically be removed while malignant tumors spread from one location to another location, thereby making them aggressive and devastating. Sarcomas are malignant tumors that arise from solid mesenchymal tissues; for example, fibrosarcoma is cancer of fibrous tissue, and lymphomas or leukemias arise from the blood mesenchymal cells [15]. Carcinomas are malignant tumors formed by epithelial cells that cover the inner and outer body surfaces. Therefore, cancers involving the ectoderm, mesoderm, or endoderm of epithelial tissue are all carcinomas irrespective of the organ in which they arise. Carcinoma can be classified into six broad groups: adrenocortical carcinoma (affects the adrenal cortex), thyroid carcinoma (affects the thyroid gland), nasopharyngeal carcinoma (affects the nose and pharynx), malignant melanoma (describes skin cancer), skin carcinoma other than melanoma, and other carcinomas including those that affect the salivary gland, lung and bronchus, colon, cervix, appendix, and urinary bladder [16]. 

The cell cycle is a normal physiological process that occurs in distinct stages in the cell to enable the proliferation of cell growth, development, and maintenance of the species. Cell proliferation and progression in normal cells are under tight genetic control. For cancer to develop, there must be a change in the genetic machinery that controls cell proliferation and eventual progression of cell cycle stages, which is the hallmark of cancer growth and sustenance [17].

Chemotherapeutic agents, targeted therapy/immunotherapy, radiation, surgery, or a combination of these treatment approaches can all be used to treat cancer, depending on the cancer stage. Chemotherapy is the center of our attention in this review. Cytotoxic agents inhibit or kill cancer cells by diverse mechanisms, some of which are well understood while others are not. Some drugs are designed to target a cancer cell’s pathophysiological survival processes, while others function as suicidal agents, altering the proliferation and metabolic activities of the cancer cells. 

Cancerous cells and normal cells share similar genetic machinery for growth and development at the cellular level. Targeting the genetic architecture of malignant cells has resulted in significant advances in the treatment of various malignancies. Some anticancer medications work by affecting cell division in rapidly diving cells, making them antiproliferative. These medications are used to stop cancer cells from progressing through the stages of the cell cycle. Some medications cause a cell arrest that is specific to a particular cell cycle stage whereas others are not. Many of them are most effective in the S-phase, causing DNA damage and triggering programmed cell death. Some block DNA synthesis either by inhibiting purine/pyrimidine synthesis or DNA polymerase inhibition. Other drugs cause direct damage by breaking DNA chains, cross-linking DNA strands, and inhibiting depolarization, while others inhibit transduction of DNA to RNA, damage mitotic spindles, or inhibit the topoisomerase enzyme [18].

Anticancer drugs used clinically based on their mechanism of action are classified into cell cycle non-specific (CCNSAs) and cell cycle specific agents (CCSAs). 

CCNSAs are drugs that are active throughout the cell cycle, e.g.,

1. Alkylating agents: nitrogen mustards (e.g., mechlorethamine, cyclophosphamide, melphalan, chlorambucil, ifosfamide, and busulfan), nitrosoureas (e.g., carmustine, lomustine and semustine, fotemustine, and streptozotocin), tetrazines (e.g., dacarbazine, mitozolomide, and temozolomide), and aziridines (e.g., thiotepa, mitomycin, and diaziquone) that deposit an alkyl group on DNA, forming a covalent bond with the nucleophilic nitrogen at position 7(N7) of guanidine or N1 and N3 of adenine and N3 of cytosine residues in DNA. This leads to an abnormal base pairing and DNA strand breakage, blockade of nucleic acid biosynthesis, direct destruction of DNA and inhibition of DNA reproduction, interference of transcription, and blockade of RNA synthesis with interference of protein synthesis and function, leading to eventual apoptosis [19,20,21], and as well influence hormone homeostasis [19].

2. Antibiotics: e.g., actinomycin that inhibits transcription by binding DNA at the transcription initiation complex and preventing elongation of the RNA chain by RNA polymerase [19].

CCSAs act at a specific phase of the cell cycle: (a) S-phase-specific drugs:

(i) Antimetabolites (e.g., antifolates, e.g., methotrexate and premetrexed, fluoropyrimidines, deoxynucleoside analogues, and thiopurines) act by inhibiting the enzyme involved in the synthesis of DNA or are incorporated as toxic analogues in the backbone of DNA during synthesis, leading to prevention of mitosis and DNA breakage [19,21] 

(ii) Topoisomerase inhibitors (two types—topoisomerase I and II). Topoisomerase I inhibitors include irinotecan and topotecan. Topoisomerase II is further divided into topoisomerase II poisons that target topoisomerase–DNA complexes, preventing DNA replication and transcription, thus, causing DNA strand breakage, and consequent apoptosis. Examples of topoisomerase II poisons are etoposide, doxorubicin, mitoxantrone, and teniposide [22]. The second group of topoisomerase II is catalytic inhibitors which block the activity of topoisomerase II, and thus, prevent DNA synthesis and translation because of the poor unwinding of the DNA. Examples of this group include novobiocin, merbarone, and aclarubicin [22]. 

(b) M-phase-specific drugs: (i) Vinca alkaloids (e.g., vincristine and vinblastine) and (ii) taxanes (e.g., paclitaxel and docetaxel) prevent cell division by preventing microtubule formation. Vinca alkaloids attach to the tubulin molecules in S-phase and hinder the proper formation of microtubules needed for M-phase [23]. They prevent the assembly of the microtubules. Additionally, podophyllotoxin is an antineoplastic that has a similar mechanism of action to *Vinca* alkaloids by binding tubulin and preventing microtubule assembly [24]. The taxanes prevent microtubule disassembly with paclitaxel acting at the G2–M border whereas docetaxel acts at S-phase. All these drugs by their activities on the microtubules prevent the cancer cells from completing mitosis and they equally have an effect on angiogenesis which is an important process that promotes tumor growth and metastasis [25]. 

(c). G2-phase-specific drugs: (e.g., bleomycin, a glycopeptide isolated from *Streptomyces verticallus*). Bleomycin intercalates DNA, and produces free radicals that harm DNA, especially when it binds to a metal ion [26,27]. 

For clarity purposes, the drugs described above are used for general treatment, and are not molecules developed from the marine sources being reviewed.

Anticancer compounds isolated from terrestrial and marine sources have a variety of mode of action for inhibiting cancer cell proliferation and/or by inducing apoptotic cell death. Dolastatin derived from *Dolabella auricularia* (a shell-less mollusk), for example, prevented cancer cells from entering the metaphase stage and triggered apoptosis in lymphoma cells [28]. By inhibiting Hsp90 in the HL60 cancer cell line, diterpene 5-episinuleptolide acetate, a non-cembranoid derived from *Sinularia* sp., triggered downstream apoptosis [29]. Flavonoids, tannins, and curcumins are polyphenolic chemicals with anticancer action [30]. Polyphenols also have antioxidant activity and are known for their apoptosis-inducing potential which they initiate by regulating the mobilization of copper ions which are bound to chromatin, causing DNA fragmentation [31]. Curcumin, a polyphenol, suppressed tumor necrosis factor (TNF) when incorporated into cancer cells in various cells lines through interaction with various stimuli [32]. Flavonoid compounds have been shown to cause cancer cell apoptosis via intrinsic and extrinsic signaling pathways, lowering mitochondrial membrane potential and inhibiting the expression of NF-κB needed for cancer cell survival, angiogenesis, and proliferation [33].

This compound 3-beta,16-beta,17-alpha-trihydroxycholest-5-en-22-one 16-O-(2-O-4-methoxybenzoyl-beta-D-xylopyranosyl)-(1-->3)-(2-O-acetyl-alpha-L-arabinopyranoside) (OSW-1) that was isolated from the bulbs of *Ornithogalum saudersiae* caused damage to mitochondrial membranes and cristae in both human pancreatic and leukemia cancer cells, with consequent loss of transmembrane potential, an increase in cytosolic calcium, and activation of calcium-dependent apoptosis [34].

The desired goal of anticancer drugs or chemotherapeutic agents is to selectively target the cancerous cells while sparing normal cells. Unfortunately, this ideal situation is far from reality because both normal and cancerous cells share the same metabolic processes, thus, normal cells are damaged. As a result, anticancer agents are not free from toxicity. The normal cells prone to damage by chemotherapy are blood-producing cells, hair follicles, and cells in the oral cavity, alimentary canal, and reproductive system [35]. The toxic effects of these drugs are numerous and include fatigue, peripheral neuropathy, nephrotoxicity, sexual dysfunction, diarrhea, and bone marrow depression, among others [35].

Development of a drug with potential anticancer activity involves preclinical screening of the product to determine its cytotoxicity on cancer cell lines. Cancer cell lines are necessary tools for cancer research, and provide a handy, cost-effective model for assessing cellular behavior and biological response. However, selection of a product as a cytotoxic substance involves its ability in inhibiting the proliferation of or killing 50% of the population of cancer cells (the IC_50_). In other words, a candidate drug that could inhibit the proliferation of or kill 50% of the population of cancer cells at a lower concentration is considered highly potent, and this relates to its intrinsic ability. Furthermore, any drug that can achieve its maximum efficacy at a much lower dose is a candidate worthy of optimization and development. Therefore, potency is a factor that is considered during preclinical screening of potential anticancer agents. From a pharmacological point of view, it is a measure of drug action expressed in relation to the amount required to produce an effect of a given intensity [36]. Drugs with very high potency produce responses at low concentrations while the less potent drugs evoke the same amount of response at very high doses. Sometimes, a less potent drug could predispose to more adverse effects than a highly potent drug because of the extended higher dose required to achieve the desired response. 

## 2. Methods

In this review, we examined papers published (in the English language only) from January 2019 to March 2020 on marine natural products or derivatives, structurally elucidated and tested for anticancer activity. The articles used were found by searching PubMed/MEDLINE and Google Scholar using Boolean operators (AND, OR, NOT) and a combination of related terms such as marine natural products or marine-derived compounds with anticancer or cytotoxic activity. Additionally, the search criteria were expanded using cross-referencing and “related articles” functions. A total of 476 articles’ abstracts were examined and those articles without isolated compounds and those with isolated compounds but not from marine sources were excluded, bringing down the total number to 195 articles. Further screening to exclude the compounds without or with less potent (IC_50_ > 20 µg/M) anticancer activity was carried out, thus, 76 articles were included in the review. From the 76 articles, we considered the IC_50_ values (≤20 µM) of 731 isolated compounds/derivatives comprising a total of 331 novel and 400 known compounds. The cytotoxic activities of these compounds were screened against a total of 120 cancer cell lines by using these assay methods: 3-(4, 5-dimethylthiazol-2-y1)-2, 5-diphenyltetrazolium bromide (MTT), Cell Counting Kit-8 (CCK-8) CellTiter-Glo (CTG), and sulforhodamide-b (SRB) assays. 

Some compounds were tested against more than one cancer cell line. However, the isolated compounds with an IC_50_ ≤ 20 µM are in the supplementary section. The structures of the novel compounds that inhibited the proliferation of some cancer cell lines with IC_50_ values ≤ 1.0 µM are shown in the text. The IC_50_ values were chosen to enable the exclusion of compounds with less potent antiproliferative activity.

## 3. Results and Discussion

### 3.1. Marine-Derived Alkaloids Cytotoxic to Cancer Cell Lines

**Figure 1:**
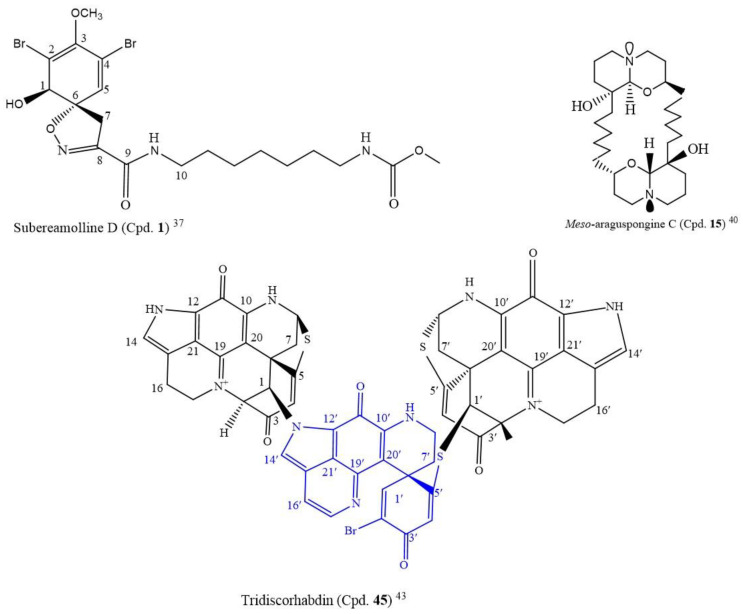
The structures of novel marine-derived alkaloid compounds cytotoxic to cancer cell lines at IC_50_ ≤ 1.0 µM.

A novel compound, subereamolline D (Cpd. **1**; Figure 1), a bromotyrosine-derived alkaloid isolated from a sea sponge *Fascaplysinopis reticulate* from the Xisha Islands, south China, inhibited the growth of the Jurkat cell line (IC_50_ of 0.88 µM) [37].

The following three new azaphilone alkaloids that contain glutamine residues, N-glutarylchaetoviridins A, B, and C (Cpds. **2**–**4**), and Cpds. **5** and **6**, which are related, were isolated from *Chaetomium globosum* HDN151398 fungal extract. N-glutarylchaetoviridins A-C (Cpds. **2**–**4**) represent the first compounds with glutamate residues. The incubation of the fungal strain with amino acids produced five azaphilone-loaded diverse amino acid residues (Cpds **7**–**11**). Thus, this method can improve the structurally diverse nature of this strain by culturing with amino acids. Unfortunately, none of these compounds exhibited a potent cytotoxic activity (IC_50_ 5.7–19.4 µM) against a panel of eleven human cancer cell lines that were screened (Table S1) when compared with a standard, adriamycin, with the range 0.1–0.6 µM IC_50_ [38].

Monanchoxymycalin C (Cpd. **12**) is a novel pentacyclic guanidine alkaloid isolated from *Monanchora pulchra*. It inhibited the proliferation of the HeLa 60 cell line with an IC_50_ of 3.5 µM (Table S1). This compound had a comparable cytotoxic effect to doxorubicin (IC_50_ = 3.26 µM) but it was more potent than cisplatin, at IC_50_ = 7.77 µM. Interestingly, monanchoxymycalin C (Cpd. **12**) had diverse modes of action, it not only induced apoptosis-related cancer cell death, but it also induced S-phase cell cycle arrest and hindered cancer cell colony formation. Additionally, it exhibited a synergistic or additive effect when combined with cisplatin, thus, making it a good prospect in cancer therapy either as a single drug or in combination with other anticancer agents [39].

Among the following nine known macrocyclic bis-quinolizidine alkaloids, araguspongine A (Cpd. **13**), araguspongine C (Cpd. **14**), *meso*-araguspongine C (Cpd. **15**) (novel), E (Cpd. **16**), L (Cpd. **17**), N (Cpd. **18**), O (Cpd. **19**), P (Cpd. **20**), petrosin (Cpd. **21**), and petrosin A (Cpd. **22**), isolated from the marine sponge *Xestospongia muta*, *meso*-araguspongine C (Cpd. **15**) and araguspongine A (Cpd. **13**) inhibited the proliferation of HepG-2, HL-60, LU-1, MCF-7, and SK-Mel-2 human cancer cells more than other compounds, with the IC_50_ range of 0.43–1.02 µM [40]. *Meso*-araguspongine is an amorphous and non-optically active solid stereoisomer of araguspongine C (Cpd. **14**). It has more cytotoxic activity to all the cancer cell lines compared to araguspongine C (Cpd. **14**) and ellipticine (positive control, IC_50_: 1.34–1.91µM) (Table S1). At a 20 µM concentration, the ten compounds also moderately inhibited the NO production in lipopolysaccharide-activated (LPS) RAW264.7 macrophages, from 7.6 to 40.8%. The structure of *meso*-araguspongine C (Cpd. **15**) is shown in Figure 1 [40].

With this IC_50_ range of 4.2–7.8 µM, the following three novel 12- or 13-membered ring macrocyclic alkaloids, ascomylactams A, B, and C (Cpds. **23**–**25**), and other known compounds phomapyrrolidone C (Cpd. **26**) and phomapyrrolidone A (Cpd. **27**), that were isolated from *Didymella* sp. CYSK-4, the mangrove endophytic fungus, moderately inhibited the growth of MDA-MB-435, MDA-MB-231, SNB19, HCT116, NCI-H460, and PC-3 human cancer cell lines, respectively (Table S1). Additionally, a single-crystal X-ray diffraction experiment was carried out on ascomylactam A and B moiety (the (6/5/6/5) tetracyclic skeletons fused with a 12- or 13-membered macrocylic ring). That was the first X-ray diffraction experiment carried out on this moiety of ascomylactam A and B [41].

HPLC-DAD-guided isolation resulted in sixteen known structurally diverse chaetoglobosins, 10-(indol-3-yl)-[13] cytochalasans (Cpds. **28**–**43**), and a new 6-O-methyl-chaetoglobosin Q (Cpd. **44**), from the coral-associated fungus *Chaetomium globosum* C2F17. However, the cytotoxicity assay on these compounds revealed that only chaetoglobosins E (Cpd. **33**) and Fex (Cpd. **38**) had significant cytotoxic activity against K562, A549, Huh7, H1975, MCF-7, U937, BGC823, HL60, HeLa, and MOLT-4 cancer cell lines with the IC_50_ range of 1.4–9.2 µM (Table S1) [42].

**Figure 2:**
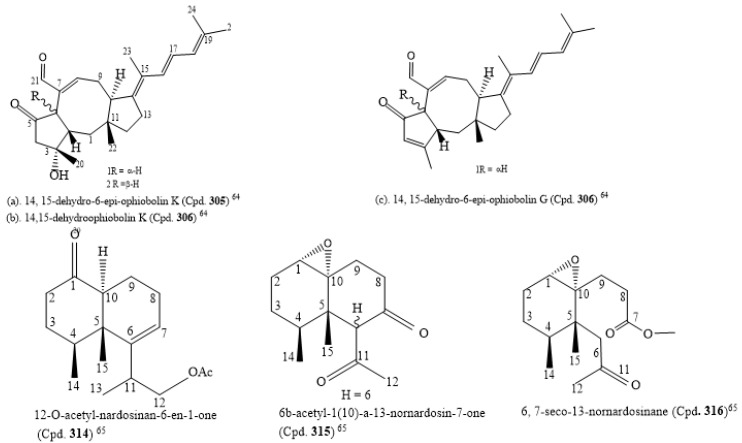
The structures of novel marine-derived terpenes and terpenoid compounds cytotoxic to cancer cell lines at IC_50_ ≤ 1 µM.

Following the LC-MS/MS molecular networking-based metabolomics and cytotoxic activity-guided study, two new discorhabdin-type alkaloids, tridiscorhabdin (Cpd. **45**) and didiscorhabdin (Cpd. **46**), were isolated from *Latrunculia biformis*, a sponge from the Weddell Sea (Antarctica). As novel compounds, a unique C-N bridge (C-1/N-13) between discorhabdin monomers was present in them, with Cpd. **45** being the first trimeric discorhabdin molecule isolated from the natural environment. Additionally, this compound was cytotoxic to human colon cancer cell line HCT 116 at 0.31 µM IC_50_. It equally inhibited the non-cancerous human keratinocyte cell line HaCaT with a similar IC_50_ value (IC_50_ = 0.92 μM), thus, suggesting its general toxicity and low selectivity for cancer cells; the structure is in Figure 2 [43].

The following four new thiodiketopiperazine alkaloids, 5’-hydroxy-6’-ene-epicoccin G (Cpd. **47**), 7-methoxy-7’-hydroxyepicoccin G (Cpd. **48**), 8’-acetoxyepicoccin D (Cpd. **49**), 7’-demethoxyrostratin C (**50**), a pair of new enantiomeric diketopiperazines, (+/−)-5-hydroxydiphenylalazine A (Cpds. **51**, **52**), and five known compounds (Cpds. **53**–**57**), were isolated from deep-sea fungus *Epicoccum nigrum* SD-388 extract. Cpds. **50** and **57** had good antiproliferative activity against Huh7.5 with the respective IC_50_ values of 9.52 and 4.88 μM. Their cytotoxic activities can be compared to that of sorafenib (IC_50_ = 8.2), the positive control, although compound 10 was more potent. The disulfide bridge at C-2/C-2’ seems to be crucial for the anticancer activity [44].

With respective IC_50_ values of 1.5, 3.8 µM for Cpd. **58** and 0.05, 0.22 µM for Cpd. **59**, streptoglutarimide H (Cpd. **65**) and known streptovitacin A (Cpd. **68**) among the new streptoglutarimides A-J (Cpds. **58**–**67**) isolated from a marine-derived actinomycete *Streptomyces* sp. ZZ741, inhibited the growth of human glioma U87MG and U251 cell lines. Streptovitacin A (Cpd. **68**) was more potent than Cpd. **65** and the positive control, doxorubicin (IC_50_: 1.6 and 6.8 µM, respectively) (Table S1) [45].

Pityriacitrin is an alkaloid of marine origin, with a typical beta-carboline scaffold. It has diverse biological functions and was isolated from Chinese *Burkholderia* sp. NBF227. A series of novel beta-carboline analogues, 9a-p (Cpds. **69**–**84**) derived from pityriacitrin, were tested for anticancer activity against the following human cancer cell lines: SGC-7901, A875, HepG2, and MARC145. Some of these beta-carboline derivatives exhibited moderate to high cytotoxic activities. Compound 9o (Cpd. **83**) with a sulfonyl group had the highest inhibitory activity against all the cell lines with the IC_50_ values of 6.82, 8.43, 7.69, and 7.19 µM, respectively. The substitution of the amide scaffold attached on the compounds was carried out with the following substituents: a phenylethylamine for compounds **9a**–**f** (Cpds. **69**–**74**), or a benzylamine for compounds **9g**–**l** (Cpds. **75**–**80**), or an arylamine for compounds **9m**–**n** (Cpds. **81**, **82**), and that did not yield analogues more active than compound **9o** (Cpd. **83**)**,** that had a sulfonyl substituent, and compound **9p** (Cpd. **84**) with an amino acid unit. Therefore, this suggested that sulfonyl group attachment on the beta-carboline scaffold is responsible for cytotoxic activity. Interestingly, some of these beta-carboline derivatives, especially **9e** (Cpd. **73**), **9l** (Cpd. **80**), **9o** (Cpd. **83**)**,** and **9p** (Cpd. **84**), were more cytotoxic on the cancer cells than the positive control, 5-fluorouracil (IC_50_ = 53.58 62. 12, 66.42, and 115.54 µM, respectively) (Table S1) [46].

The fermentation of broth of the coral-associated *Aspergillus ochraceus* strain LCJ11-102 led to the isolation of four new ochrazepine A, B, C, and D (Cpds. **85**–**88**) conjugates. By the nucleophilic addition to the epoxide, these conjugates dimerized from 2-hydroxycircumdatin C (Cpd. **89**) and aspyrone (Cpd. **90**) and by the semisynthesis involving a nucleophilic addition of 2-hydroxycircumdatin C (Cpd. **89**) to aspyrone (Cpd. **90**), Cpds. **85**–**88** were obtained. Cpd. **85** was cytotoxic to ten human cancer cell lines, Cpds. **86** and **88** were selectively cytotoxic to the U251 cell line, and Cpd. **87** was active against A673, U87, and Hep3B cell lines with IC_50_ values of 2.5–11.3 µM among the 26 screened human cancer cell lines (Table S1) [47].

### 3.2. Marine-Derived Terpenes and Terpenoids Cytotoxic to Cancer Cell Lines

*Eutypella* sp. D-1, an arctic fungus that produces cytotoxic cyclopropyl- and cyclobutyl-fused pimarane diterpenoids when grown on a conventional medium, was stimulated to produce extracts that gave rise to four new meroterpenoids, eutypellacytosporins A–D (Cpds. **91**–**94**), as well as the known biogenetically related compound cytosporin D (Cpd. **95**) when grown on rice medium. Cpds. **91**–**94** contain 12, 32-ester linkage of cytosporin D (Cpd. **95**), and decipienolide A or B moieties. The new compounds had weak antiproliferative activity against DU145, SW1990, Huh7, and Panc-1 cell lines with the IC_50_ range of 4.9 to 17.1 µM (Table S2) [48].

The isolation of marine compounds from *Aspergillus versicolor* ZZ761 resulted in a new compound, indoloditerpene (Cpd. **96**), and fifteen known Cpds. (**97**–**111**). These compounds were tested for anticancer activity, but only diorcinol (Cpd. **97**) and versicolorin B (Cpd. **101**) had antiproliferative activity against human glioma U87MG and U251 cell lines with IC_50_ values of 4.4 and 6.2 µM and 11.3 and 30.5 µM, respectively (Table S2) [49].

From the Vietnamese marine sediment-derived fungus *Aspergillus flocculosus,* four new compounds, one aspyrone-related polyketide aspilactonol G (Cpd. **112**), one meroterpenoid 12-epi-aspertetranone D (Cpd. **113**), two drimane derivatives (Cpds. **114**, **115**), and five known Cpds. **116**, **117**, **118**, **119**, **120** were isolated. These compounds were screened for anticancer activity against the human prostate cancer cell line 22Rv1, human breast cancer cell line MCF-7, and murine neuroblastoma Neuro-2-A cell line. However, only insulicolide A (Cpd. **114**) exhibited cytotoxic activity against 22Rv1 and Neuro-2-A cell lines at IC_50_ values of 3.0 and 4.9 µM, respectively, that are below 20.0 µM (Table S2) [50].

Yu et al. isolated three new sesquiterpene quinones/hydroquinones, 20-demethoxy-20-isopentylaminodactyloquinone D (Cpd. **121**), 20-demethoxy-20-isobutylaminodactyloquinone D (Cpd. **122**), and 19-methoxy-dictyoceratin-A (Cpd. **123**), and five known related compounds (Cpds. **124**–**128**) from the marine sponge *Dactylospongia elegans*. These compounds belong to the meroterpenoid class of terpenes. The cytotoxicity assay on the compounds against the human cancer cell lines DU145, SW1990, Huh7, and Panc-1 revealed growth inhibitory activity on the cell lines with IC_50_ values in the range of 2.33–37.85 µM. Thus, only the compounds with IC_50_ below 20.0 µM are in Table S2 [51].

A deep-sea-derived fungus *Penicillium allii-sativi* gave rise to two new (andrastane A (Cpd. **129**), 16-epi-citreohybriddione A (Cpd. **130**)) and one known (Cpd. **131**) meroterpenoids. Cpd. **129** is a rare meroterpene without the lactone moiety but possesses the cyclopentan-1, 3-dione group. The three compounds were screened for their antiproliferative activity against HepG2, A549, BIU-87, BEL-7402, ECA-109, HelaS3, and Panc-1 cancer cells. Indeed, only Cpd. **129** demonstrated cytotoxic activity with a selective growth inhibition against HepG2 (IC_50_ = 7.8 µM). However, the study on the mechanism of action of Cpd. **129** showed that it induced apoptosis in HepG2 cells via direct caspase-8-mediated caspase-3 activation. Additionally, a dual luciferase reporter gene assay carried out to investigate the retinoid X receptor alpha (RXRα) transcriptional activity revealed that Cpd. **129** increased the reporter transcriptional activation of RXRα and reduced the trans-activity of RXRα induced by 9-cis-RA [52].

By using chromatographic separation techniques, sixteen known compounds (Cpds. **133**–**148**), including one new triterpene saponin, aegicoroside A (Cpd. **132**), were isolated from Vietnamese mangrove *Aegiceras corniculatum* leaves. The compounds were tested for cytotoxic activity against MCF-7 (breast), HCT116 (colon), B16F10 (melanoma), and A549 (lung adenocarcinoma) cancer cell lines. Sakurasosaponin (Cpd. **133**) inhibited the proliferation of the four cancer cell lines screened, and sakurasosaponin methyl ester (Cpd. **134**) inhibited the growth of MCF-7, A549, and HCT116 cell lines with IC_50_ values ranging from 2.89 to 9.86 µM (Table S2) [53].

Two novel capnosane-based diterpenoids, flaccidenol A (Cpd. **149**) and 7-epi-pavidolide D (Cpd. **150**), two new cembranoids, flaccidodioxide (Cpd. **151**) and flaccidodiol (Cpd. **152**), and three known compounds, sarcophytol T (Cpd. **153**), flaccidoxide-13-acetate (Cpd. **154**), and 14-*O*-acetylsarcophytol B (Cpd. **155**) were sourced from *Klyxum flaccidum*, a marine-soft coral from the island of Pratas. That is the first time the novel capnosane diterpenoids were isolated from the *Klyxum* genus. The cytotoxicity of Cpds. **149** to **155** against the proliferation of human lung adenocarcinoma (A549), human colorectal adenocarcinoma (DLD-1), and mouse lymphocytic leukemia (P388D1) cell lines were assayed. Cpds. **149** and **155** inhibited the growth of all the cancer cells in the range of 6.0-11.7 µM, but Cpd. **151** inhibited the growth of only P388D1 (IC_50_ = 19.6 µM) (Table S2). Besides being cytotoxic, Cpds. **149** and **155** exhibited anti-inflammatory activity against the N-formyl-methionyl-leucyl-phenylalanine/cytochalasin B (fMLF/CB)-stimulated human neutrophils through the suppression of superoxide anion generation and elastase release. Furthermore, Cpd. **155** effectively inhibited the release of elastase, and it is worthy of mention that this is the first report of the biological activities of Cpd. **155 [54]**.

Long-term research on the Taiwanese soft coral *Asterospicularia laurae*, conducted by Su et al., led to the isolation of Xenicane-type diterpenoids such as asterolaurins A-M from *A. laurae* coral tissues during the non-spawning period. Recently, the authors isolated a new xenicane diterpenoid, asterolaurin N (Cpd. **156**), along with three known xenicane-type monocarbocyclic diterpenes (13-epi-9-desacetylxenicin (Cpd. **157**), xeniolide-B 9-acetate (Cpd. **158**), and asterolaurin I (Cpd. **159**)) from *A. laurae* during the spawning period. The compounds were screened for cytotoxic activity against Molt 4, K562, Sup-T1, and U937 human cancer cell lines. Cpds. **156**, **157**, and **158** were selectively cytotoxic to Molt 4, and Cpd. **157** to Molt 4, K562, Sup-T1, and U937 cell lines (Table S2) [55]. Further studies to elucidate the structure of the new compound showed that the C-15 of Cpd. **156** contains two methyl groups on a carbon-bearing acetyl group, and this has not been reported previously [55].

Three new cembranolides (Cpds. **160**–**162**) and six known cembranolide diterpenoids (Cpds. **163**–**168**) were isolated from an Okinawan soft coral *Lobophytum* sp. The new compounds were screened for anticancer activity against HeLa, A459, B16-F10, and RAW 264.7 cells and anti-inflammatory effect in LPS-stimulated inflammatory RAW 264.7 macrophage cells. The result revealed that only cembranolide diterpene derivative 1 (Cpd. **160**) was antiproliferative against the cancer cells below 20 µM IC_50_ (Table S2) and anti-inflammatory (IC_50_ = 7.75 µM) by the inhibition of nitric oxide production in the LPS-induced RAW 264.7 macrophage cells, more than the other compounds. Further studies on structural elucidation revealed all the compounds contain an alpha-methylene-gamma-lactone ring adjacent to a cembrane and Cpds. **160**, **165**, **156,** and **157** have an epoxide ring that may be responsible for the mild bioactivities of Cpd. **160** [56].

From the methanol extract of *Spongia tubulifera*, a sponge sourced from the Mexican Caribbean, two new spongian furanoditerpenes, 3-beta-hydroxyspongia-13(16),14-dien-2-one (Cpd. **169**) and 19-dehydroxy-spongian diterpene 17 (Cpd. **170**), along with five known terpenes, the spongian furanoditerpenes-9-nor-3-hydroxyspongia-3,13(16),14-trien-2-one (Cpd. **171**), 3-beta-19-dihydroxyspongia-13(16), 14-dien-2-one (epispongiadiol) (Cpd. **172**) and spongian diterpene 17 (Cpd. **173**), the furanoditerpene ambliol C (Cpd. **174**), and the sesterterpene scalarin (Cpd. **175**), were isolated. Cpds. **169**, **171**, and **174** displayed weak cytotoxic activity against MCF-7 and Mia Paca-2 human cancer cell lines. However, only Cpd. **174** inhibited the growth of MCF-7 and Mia Paca-2 at an IC_50_ below 20 µM (Table S2) [57].

Aphidicolin is a DNA polymerase alpha inhibitor that has been investigated in clinical trials for the treatment of cancer. In recent times, researchers have discovered about 300 modified aphidicolins, but unfortunately, none has shown a significant biological effect. Nonetheless, 71 new aphidicolins A**176**–A**246** (Cpds. **176**–**246**) and eight known (Cpds. **247**–**254**) aphidicolin congeners from a fungus *Botryotinia fuckeliana* MCCC 3A00494 from the western Pacific Ocean (−5572 m), were reported by Niu et al. The structures of Cpds. **176**–**246** were determined by spectroscopic analysis, X-ray crystallography, chemical derivatization, modified Mosher’s method, and the ECD exciton chirality method. Cpds. **229**–**232** and **233**–**239** are novel 6/6/5/6/5 pentacyclic aphidicolins with tetrahydrofuran and dihydrofuran rings, respectively, and Cpds. **240**–**246** are rare noraphidicolins. Aphidicolin A183 (Cpd. **183**) induced apoptosis in T24 (IC_50_ = 2.5 µM) and HL-60 (IC_50_ = 6.1 µM) cancer cells (Table S2) by causing DNA damage. With the docking of its molecular structure to the human DNA polymerase alpha binding pocket, Cpd. **183** formed tight intermolecular contacts, substantiating aphidicolin A8 as a potent cytotoxic lead compound [58].

The treatment of Hawaiian marine sponge *Dactylospongia elegans* extracts led to the isolation of nine known sesquiterpenoid quinones and quinols (Cpds. **255**–**263**), and kauamide (Cpd. **263**), a new polyketide-peptide containing an 11-membered heterocycle. From the spectroscopic analyses, the planar structure of Cpd. **263** was determined; density functional theory (DFT) calculations of the GIAO NMR shielding tensors and advanced Marfey’s analysis of the N-MeLeu residue were used to determine its relative and absolute configurations, respectively. Cpds. **255** and **257** had moderate inhibitory activity against beta-secretase 1 ((BACE1) IC_50_ > 20 µM), whereas Cpds. **255**–**263** exhibited mild to potent antiproliferative effects on human glioma (U251) cells (Table S2). Cpds. **255**, **256**, and **258**–**261** were also active against human pancreatic carcinoma (Panc-1) cells (Table S2). Unfortunately, kauamide (Cpd. **263**) did not show any significant biological activity in the two assays (60% inhibition at 50 µM against U251; 15% inhibition at 83 µM against BACE1), although it was not tested against Panc-1 [59].

A good source of erythrolide-chlorinated briarane diterpenoids could be traced to the Caribbean soft coral *Erythropodium caribaeorum*. The ecological role of these compounds as feeding deterrents is a fact. They have a wide variation in their composition based on the location of the sample collection. This soft coral can be found in many locations of the Caribbean Sea in Colombia, including Santa Marta, Islas del Rosario, and Providencia, which make up several coral reef zones in the south and southwest Caribbean Sea. The authors evaluated the differences in erythrolide composition with the metabolic profiles of the samples collected from each of these locations by HPLC-MS analyses. The principal component analysis showed a difference in the diterpene composition according to the origin of the sample collected. In addition, diterpenes from the collected samples from each location were isolated to describe the three chemotypes. Thus, the chemotype from Santa Marta was highly varied, with new erythrolides W (Cpd. **265**) and X (Cpd. **266**) together with eight known erythrolides (Cpds. **267**–**274**). The sample from Islas del Rosario had a low-diversity chemotype constituted by large amounts of erythrolide A (Cpd. **275**) and B (Cpd. **276**). The chemotype from Providencia had low chemical diversity with only two compounds, erythrolide V (Cpd. **277**) and R (Cpd. **278**). The evaluation of the cytotoxic activity of the compounds against the human cancer cell lines PC-3, MCF-7, and A549 revealed erythrolides A (Cpd. **275**), B (Cpd. **276**), and D (Cpd. **268**) as the more active compounds with IC_50_ values in the range of 2.45–30 µM (Table S2) [60]. Notably, erythrolides R and V (Providencia Island chemotype bearing a free OH in C-5) did not exhibit any cytotoxicity, suggesting the role of the acetyl group at C-5 in the recorded activity.

A broad phytochemical analysis of *Schisandra sphenanthera* leaves produced six highly oxygenated nortriterpenoids (Cpds. **279**–**284**), five lignans (Cpds. **285**–**289**), one new pre-schisanartane-type schisandrathera A (Cpd. **285**), a new dibenzocyclooctadiene glycoside, schisandrathera B (Cpd. **286**), and two new lignans, schisandrathera C (Cpd. **287**) and schisandrathera D (Cpd. **288**). Their chemical structures and absolute configurations were determined by HR-ESI-MS, NMR, and ECD spectra. Additionally, the isolated compounds were tested for anticancer activity against PC3 (prostate cancer) and MCF-7 (breast cancer) cell lines. Amongst the compounds, henridilactone A (Cpd. **281**) and schirubrisin B (Cpd. **289**) had equal cytotoxic potency on PC3 with the same IC_50_ value of 3.21 µM, and the remaining nine compounds were less active in the screened models (Table S2) [61].

A new sesquiterpene, (+)-19-methylaminoavarone (Cpd. **290**), and six known compounds (Cpds. **291**–**296**) were isolated from the *Dysidea* sp., a marine sponge from the Xisha Islands. There was a revision of the carbon spectrum data of Cpds. **291,** and the absolute configurations of Cpds. **290** and **291** were confirmed by electronic circular dichroism (ECD) analysis. Cpds. **290**–**292** and **294**–**296** had moderate to good cytotoxic activity against several human cancer cell lines (Table S2) [62].

Penicindopene A (Cpd. **297**), a new indole diterpene, together with seven known compounds (Cpds. **298**–**304**), became isolated from the deep-sea fungus *Penicillium* sp. YPCMAC1. From the structural analysis studies, penicindopene A was the first example of an indole diterpene that possesses a 3-hydroxyl-2-indolone moiety; it was moderately cytotoxic to A549 and HeLa cell lines with IC_50_ values of 15.2 and 20.5 µM, respectively [63].

The isolation of five new sesterterpenes, 14,15-dehydro-6-*epi*-ophiobolin K (Cpd. **305**), 14,15-dehydroophiobolin K (Cpd. **306**), 14,15-dehydro-6-*epi*-ophiobolin G (Cpd. **307**), 14,15-dehydro-ophiobolin G (Cpd. **308**), and 14,15-dehydro-(*Z*)-14-ophiobolin G (Cpd. **309**), along with four known ophiobolins (Cpds. **310**–**313**), was carried out with *Aspergillus flocculosus*, a marine fungus sourced from the seaweed *Padina* sp. of Vietnamese origin. The five new ophiobolins were first isolated as ophiobolin derivatives that had a fully unsaturated side chain. Their structures were elucidated using 1D, 2D NMR, and HR-ESIMS spectroscopic methods. The absolute configurations were determined by the comparison of chemical shifts and optical rotation values with those of known ophiobolins. All compounds (Cpds. **310**–**318**) were screened for cytotoxicity against six cancer cell lines, HCT-15, NUGC-3, NCI-H23, ACHN, PC-3, and MDA-MB-231, and they were potently cytotoxic to the cancer cell lines with an IC_50_ value range of 0.14 to 2.01 µM (Table S2). The consideration of the structure–activity relationship of the compounds revealed that ophiobolins 14, 15-dehydro-6-*epi*-ophiobolin K (Cpd. **310**) and 14,15-dehydroophiobolin K (Cpd. **306**) have three double bonds at their side chain and are stereoisomers with A/B *trans*-ring and A/B *cis*-ring structures, respectively, while the known compounds (Cpds. **310**–**312**) had one double bond. Thus, this may be the reason why Cpd. **308** had weaker activity than the known ophiobolins (Cpds. **310**–**311**), and also why 14, 15-dehydro-6-*epi*-ophiobolin K (Cpd. **307**) had the highest antiproliferative activity against the breast cancer cell line compared to adriamycin, the positive control (IC_50_ of 0.15 µM). Cpd. **309** (IC_50_ of 1.75 µM, Table S2) had the weakest cytotoxic activity against the cell line MDA-MB-231. These findings on ophiobolins suggest that the C-14/C-15 geometry might affect their activity. Additionally, the C-6 stereochemistry and the hydroxyl group at C-3 may not significantly affect the cytotoxicity [64]. The structures of the new compounds with IC_50_ below 1.0 µM are in Supplementary Material citation [64].

**Figure 3:**
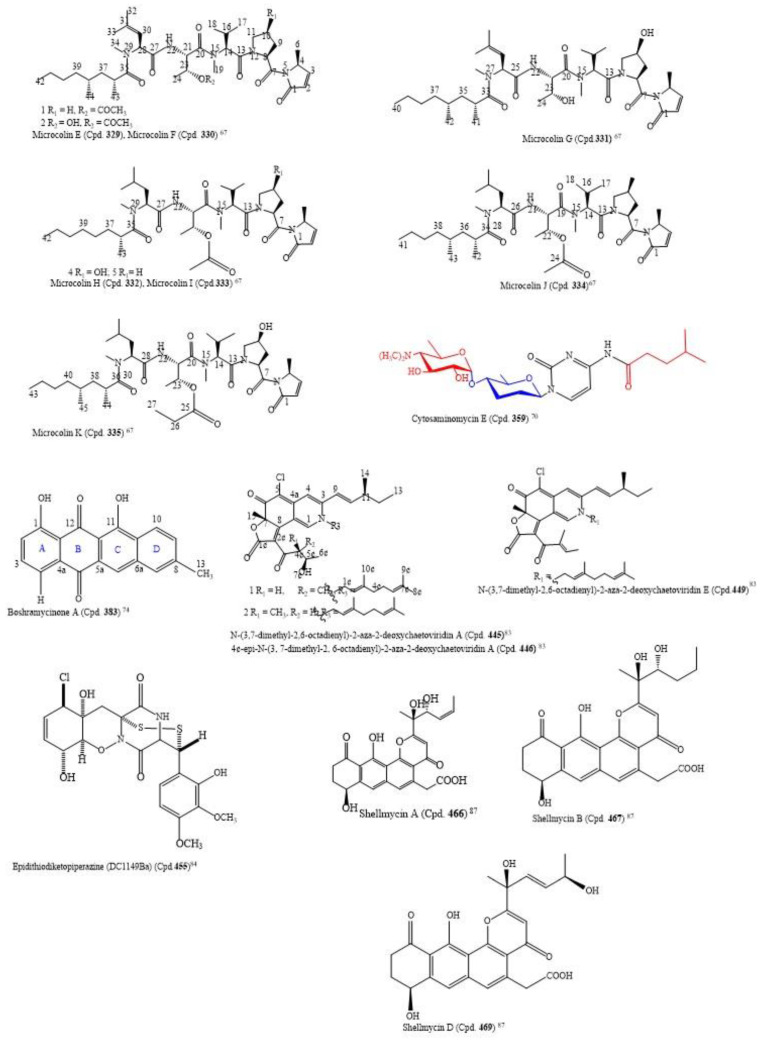
The structures of novel marine-derived amino acid, peptide, and polyketide compounds cytotoxic to cancer cell lines at IC_50_ ≤ 1 µM.

Three novel nardosinane-type sesquiterpenes, 12-O-acetyl-nardosinan-6-en-1-one (Cpd. **314**), 6b-acetyl-1(10)-a-13-nornardosin-7-one (Cpd. **315**), and 6, 7-seco-13-nornardosinane derivatives (Cpd. **316**), together with six known compounds (Cpds. **317**–**322**), were isolated from *Rhytisma fulvum fulvum*, an alcyonacean soft coral. Due to the C-6 epimerization of Cpd. **317**, Cpd. **315** was considered an artifact. All the compounds were cytotoxic to NCI-H1299, HepG2, and MCF-7 with IC_50_ ranges of 0.0352–0.0974, 0.0717–0.3745, and 0.0341–0.1325 µM, respectively (Table S2). The structures of the novel compounds are shown in Figure 3 [65].

### 3.3. Marine-Derived Amino Acids, Peptides, and Polyketides Cytotoxic to Cancer Cell Lines

The following four new polyketide ansamycins: divergolides (macrolides) T, U, V, and W (Cpds. **323**–**326**) and two known analogues (Cpds. **327** and **328**) remained isolated from the fermentation broth of the mangrove-derived actinomycete *Streptomyces* sp. KFD18. By using spectroscopy and single-crystal X-ray diffraction analyses, their structures and the absolute configurations of their stereogenic carbon were determined. Cpds. **323**–**326** exhibited cytotoxic activity against the human gastric cancer cell line SGC-7901, the human leukemic cell line K562, the cervical cell line HeLa, and the lung carcinoma cell line A549. Cpd. **325** was the most active; Cpds. **327** and **328** were inactive against all the tested cancer cell lines (Table S3). Cpds. **323** and **325** had potent and specific cytotoxic activity against the SGC-7901 cells (IC_50_ 2.8 and 4.7 µM, respectively). They were more cytotoxic to the cell line than the two positive controls, imatinib (IC_50_ = 86.8 µM) and adriamycin (IC_50_ = 6.9 µM), used in the study. They induced apoptosis in SGC-7901 cells after double-staining with acridine orange–ethidium bromide (AOEB) and 4’, 6-diamidino-2-phenylindole (DAPI). This is the first time the apoptosis-inducing potential of divergolides is reported [66].

By using the bioactivity-guided study and the LC-MS/MS molecular networking approach, the following nine new linear lipopeptides: microcolins E, F, G, H, I, J, K, L, and M (Cpds. **329**–**337**) and four known related compounds, microcolins A, B, C, and D (Cpds. **338**–**341**) were isolated from *Moorea producens*, a marine cyanobacterium. Catalytic hydrogenation of Cpds. 338–341 produced two known compounds, 3,4-dihydromicrocolins A and B (Cpds. **342**, **343**), and two new derivatives, 3,4-dihydromicrocolins C and D (Cpds. **344**, **345**), respectively. The combination of spectroscopic and advanced Marfey’s analysis was used to determine the structures of the new compounds. Surprisingly, in Cpds. **329**–**341** and **336**, structurally unusual amino acid units, 4-methyl-2-(methylamino)-pent-3-enoic (Mpe) acid and 2-amino-4-methylhexanoic acid (N-Me-homoisoleucine), respectively, were discovered; these are rare residues in naturally occurring peptides. The analogues had cytotoxic activity against H-460 human lung cancer cells at very low IC_50_ values ranging from 0.037–5.0 µM (Table S3). The structures of the compounds with IC_50_ ≤ 1.0 µM are in Figure 3 [67]. Thus, the structure–activity relationship found by the authors to compare the relative cytotoxic potencies of the compounds revealed that a hydroxyl group at C-4 of Pro and a double bond in the Mdp moiety are crucial for activity. This is an observation in tandem with the findings of [68]. Additionally, the loss of the N-methyl group from the Val residue as found in microcolin M (Cpd. **337**) increased cytotoxic activity from 0.910 µM in microcolin B (Cpd. **339**) to 0.069 µM in microcolin J (Cpd. **334**), consequently increasing the potency of the analogue. In addition, the presence of an acetyl group at C-3 of Thr, for instance, by comparing microcolin F (Cpd. **330**) with microcolin G (Cpd. **331**) and microcolin A (Cpd. **338**) with microcolin D (Cpd. **341**; IC_50_ 0.075 µM), increased the cytotoxicity. The propionate group at the C-3 position of Thr decreased the cytotoxicity (microcolin K (Cpd **335**), which is about 32-fold less potent than microcolin A). Likewise, adding a double bond in the M-ME-Leu residue of microcolins E-G (Cpds. **329**–**341**) led to the loss of the antiproliferative activity. Moreover, the removal of one of the pendant methyl groups in the fatty acid chain does not affect the cytotoxic activity significantly; thus, it does not seem to play a role in determining the cytotoxic activity of the series [67].

Deep-sea actinomycete *Nonomuraea* sp. AKA32 produced a new and two known aromatic polyketide cytotoxic compounds that inhibited the growth of a murine B16 melanoma cancer cell line. The new compound is akazamicin (Cpd. **346**), and the two known compounds are actinofuranone C (Cpd. **347**) and N-formylanthranilic acid (Cpd. **353**). The cytotoxic IC_50_ values of the compounds were 1.7, 1.2, and 25 µM, respectively [69].

From marine-derived *Streptomyces* sp. SSA28, eleven new pyrimidine nucleosides (Cpds. **349**–**359**) and 12 known derivatives (Cpds. **360**–**371**) were isolated (Table S3). Cpds. **35**–**364** were cytotoxic to the human colon cancer cell line HCT-116 with IC_50_ values from 0.39–6.63 µM. The structure of cytosaminomycin E (Cpd. **359**) is in Figure 3 [70].

Out of the four new cycloheptapeptides, fuscasins A-D (Cpds. **372**–**375**) that were isolated from the marine sponge *Phakellia fusca* from the South China Sea, fuscasin A (Cpd. **372**), that bears a pyrrolidine-2, 5-dione, had potent cytotoxic activity. It inhibited the growth of only HepG2 (IC_50_ = 4.6 µM) from the six cancer cell lines (MCF-7, HeLa, NCI-H460, PC9, and SW480) that were screened. Interestingly, it did not inhibit the growth of cardiomyoblast H9C2, a normal cell line (IC_50_ value of 100 µM), which suggests that Cpd. **372** may exhibit selective toxicity only on the cancer cell while sparing the normal cells. The planar structures of the compounds were characterized by using spectroscopic methods, and the advanced Marfey’s method was used to determine the absolute configurations of amino acid residues [71].

A new monomeric julichrome, julichrome Q10 (Cpd. **376**), five known julichrome derivatives, julichrome Q6 (Cpd. **377**), julichrome Q6.6 (Cpd. **378**), julichrome Q3.5 (Cpd. **379**), julichrome Q5.6 (Cpd. **380**), and julichrome Q2.3 (Cpd. **381**), and gliotoxin (Cpd. **382**), a diketopiperazine, were isolated from *Streptomyces* sp. derived from soil. The cytotoxic activity of the compounds was investigated against human hepatocarcinoma HepG-2 and SMMC-7721 cell lines, human breast cancer MCF-7 and MDA-MB-231 cell lines, and the human non-cancerous hepatic cell line (L-02). Gliotoxin (Cpd. **382**) exhibited the most cytotoxic activity against the screened tumorigenic cell lines with IC_50_ values in the range of 0.11 to 1.45 µM. The gliotoxin was more cytotoxic to the cell lines than the positive control, doxorubicin (IC_50_ = 1.37, 0.36, 0.48, 0.94, 1.13 µM), except to HepG-2. Julichrome Q6.6 (Cpd. **378**) was selectively cytotoxic to SMMC-7721, MCF-7, and MDA-MB-231 cell lines while sparing the L-02 (Table S3) [72].

The culture broth of the marine-derived *Streptomyces* sp. Mei 16-1, 2 produced three new anthracyclinones, boshramycinones A–C (Cpds. **383**–**385**), 2-acetyl-1, 8-dihydroxy-3-methyl-anthraquinone (Cpd. **386**), bafilomycin B1 (Cpd. **387**), bafilomycin B2 (Cpd. **388**), and C1-amide (Cpd. **389**). The isolated compounds and the crude extract were assayed for cytotoxic activity against 36 human cancer cell lines (Table S3).

Boshramycinone B (Cpd. **384**) exhibited a weak and non-selective cytotoxic effect on the 36 human cancer cell lines with a mean IC_50_ value of 38.30 µM/mL [73]. Amazingly, boshramycinone A (Cpd. **383**) was appreciably more potent than Cpd. **384** with an IC_50_ range of 0.51–17.27 (Table S3) and a mean IC_50_ of 1.75 µM/mL [74]. It had a moderate selectivity index across the 36 cancer cell lines that were tested, with the 22Rv1 cell line achieving an individual IC_70_ at <1/3 the mean IC_70_ (<1.55 µM/mL) and other 13 cell lines achieving individual IC_70_ values at <1/2 the mean IC_70_ (<2.32 µM/mL). Marked selectivity is considered a sign of tight binding, and not as a result of a general cytotoxicity [74]. The structure of boshramycinone A (Cpd. **383**) is in Figure 3.

The isolation from the fungus *Penicillium citrinum* SCSIO 41017 linked with the sponge *Callyspongia* sp. yielded two new polyketides, xerucitrinin A (Cpd. **390**) and coniochaetone M (Cpd. **391**), one new steroid, 16-α-methylpregna-17α, 19-dihydroxy-(9, 11)-epoxy-4-ene-3,18-dione-20-acetoxy (Cpd. **392**), and eleven known analogues (Cpds **393**, **394**, **395**, **396**, **397**, **398**, **399**, **400**, **401**, **402**, **403**). The bioactivity studies on the compounds revealed Cpd. **396** as a cytotoxic agent against the MCF-7 cell line with 1.3 µM IC_50_, which is more potent than taxol (1.4 l µM). However, Cpd. **392** exhibited moderate activity against all cell lines with an IC_50_ range of 13.5–18.0 µM [75].

Three new versiol-type derivatives, named peniciversiols A–C (Cpds. **404**–**406**), two novel lactone derivatives (penicilactones A and B (Cpds. **407** and **408**)), and 11 known polyketides (Cpds. **408**–**419**) were isolated from the deep-sea-derived fungus *Penicillium chrysogenum* MCCC 3A00292 after the chemical treatment of the solid cultures. Cpd. **405** has a 2, 3-dihydropyran-4-one ring; it is the second example of a versiol with this ring structure. Moreover, Cpds. **404** and **410** were the first lactone derivatives with the *α*-methyl-*γ*-hydroxy-*γ*-acetic acid *α*, *β*-unsaturated-*γ*-lactone moiety and *α*-hydroxy-*γ*-methyl-*γ*-acetic acid *α*, *β*-unsaturated-*γ*-lactone unit, respectively. These compounds were screened for antiproliferative activities against five human malignant cell lines, comprising BIU-87, ECA109, BEL-7402, Panc-1, and HeLa-S3. There was a selective antiproliferative effect against BIU-87 by Cpd. **409** with 10.21 µM IC_50_. Additionally, Cpds. **407**, **408**, **411**, and **415**–**419** inhibited the growth of ECA109, BIU-87, and BEL-7402 cell lines with the IC_50_ range of 7.70–20 µM (Table S3) [76].

The isolation of sekgranaticin (Cpd. **420**), granaticins A (Cpd. **421**) and B (Cpd. **422**), and methyl granaticinate (Cpd. **423**), was carried out with the culture broth of *Streptomyces* sp. 166#. Sekgranaticin (Cpd. **420**) is a novel hybrid polyketide with a complex 6/6/6/6/6/6/6 7-ring system [77]. The compounds inhibited the growth of MCF-7, A549, P6C, and HCT-116 cancer cell lines with the IC_50_ values of 0.02−6.77 μM (Table S3) [77].

A new polyene compound (Cpd. **424**) and a new diketopiperazine (Cpd. **425**), in addition to three known compounds (Cpd. **426**–**428**), were isolated from *Penicillium crustosum* HDN153086, an Antarctic marine-derived fungus. The structures of Cpds. **424**–**428** were deduced from the MS, NMR, and TD-DFT calculations of specific ECD spectra. The compounds were tested for cytotoxic activities against the K562 cell line. However, only Cpd. **425** (fusaperazine F) is cytotoxic to cancer cells, with an IC_50_ of 12.7 μM [78].

Polyether compounds from *Streptomyces* species are known for antibacterial, antiviral, antiparasitic, antifungal, and antitumor activities. Some of these compounds target cancer stem cells and multi-drug-resistant cancer cells [79]. The authors isolated three polyether-type metabolites (Cpds. **429**–**431**) from the marine-derived *Streptomyces cacaoi* through antimicrobial bioactivity-guided fractionation. Cpd. **431** is a new natural product. As several polyether compounds with structural similarities, such as monensin, have been associated with autophagy and cell death, they assessed the cytotoxicity of these three compounds. From the cytotoxicity screening, Cpds. **430** and **431** were cytotoxic to human cancer cell lines CaCo-2, HeLa, PC-3, and A549, but not to non-cancerous human cell lines MRC-5 and Vero. Unfortunately, the cytotoxic activity of compound **429** was not determined. Cpds. **430** and **431** were selective for CaCo-2 and PC-3 at a low IC_50_ (7.4 and 11.8 µM for Cpd. **430**), respectively (Table S3). In addition, Cpds. **430** and **431** induced the accumulation of autophagy flux markers, LC3-II and p62, and caused the cleavage of caspase-3, caspase-9, and poly (ADP-ribose) polymerase 1 (PARP-1). There was a dose-dependent down-regulation of the proteins that mediate the autophagosome by the compounds. This study provided an insight into the molecular mechanisms of the polyether-type polyketides and suggests their potency as inhibitors of autophagy and apoptosis inducers [79].

Five novel anthraquinone derivatives, auxarthrols D, E, F, G, and H (Cpds. **432**–**436**), and two known compounds (Cpds. **437**, **438**), were obtained from marine-derived fungus *Sporendonema casei* culture. However, Cpd. **436** was the second isolated anthraquinone derivative that has a chlorine atom. The seven compounds were screened for anticancer activity against ten human cancer cell lines, HL-60 Hela, HCT-116, MGC-803, HO8910, MDA-MB-231, SH-SY5Y, PC-3, BEL-7402, and K562, and a non-cancer cell line, L-02. Cpds. **432** and **434** had cytotoxic activities with IC_50_ values from 4.5 µM to 22.9 µM (Table S3) [80]. Cpds. **435** and **437** were the first reported anthraquinone derivatives with an anticoagulant property. They had an inhibition ratio of 47.8% and 51.5%, respectively, against the positive control, argatroban (inhibition ratio: 65.0%).

From *Jaspis splendens*, an Indonesian marine sponge, the following compounds, Jasplakinolide (Cpd. **439**) (the parent compound), a new acylic jasplakinolide congener (Cpd. **440**), an acyclic derivative (Cpd. **441**) that requires revision (Cpd. **442**), and two jasplakinolide derivatives (Cpds. **443, 444**), were isolated. The chemical structures of the new and known compounds were elucidated based on HRESIMS and 1D and 2D NMR spectral analysis. The NMR data of the other compounds were compared with those of jasplakinolide (Cpd. **439**). The compounds inhibited the proliferation of mouse lymphoma (L5178Y) cells with nanomolar to micromolar IC_50_ values (Table S3). (+)-Jasplakinolide Z6 (Cpd. **440**) is an acyclic derivative of Cpd. **441**, lacking the depside bond between C-1 and C-15, and with an IC_50_ of 3.2 µM, it was the least potent derivative compared to Cpds. **439**, **442**, and **443,** whose IC_50_ values were in the nanomolar range (<100 nM), but it was more potent than the positive control, kahalalide F (IC_50_ = 4.3 µM) [81]. This shows that the 19-membered ring of jasplakinolide is not the main structural requirement for its activity. Therefore, lipophilicity influences the antiproliferative activities of the acyclic jasplakinolide congener. This is because of free carboxylic acid functionality in Cpd. **440** that imparts higher polarity to it more than Cpd. **442**, thus rendering Cpd. **440** less lipophilic. Additionally, further proof from previous research [81] on the antiproliferative activity of three acyclic jasplakinolides Z1–Z3 against a panel of human cancer cells revealed that jasplakinolides Z2 and Z3 derivatives were the methyl and ethyl esters of jasplakinolide Z1, respectively. Results also show that the hydrolysis of the depside bond yields a free carboxylic acid that diminished its activity significantly, which reverted to being equivalent to the parent compound by converting the carboxylic acid group into ester functionality. That may be due to increased lipophilicity of the ester derivatives that may facilitate their cellular uptake and hence recover the activity of the compound [82].

The isolation of the eight new nitrogenated azaphilones (Cpds. **445**−**452**) and two known compounds (chaetoviridin A (Cpd. **453**) and chaetoviridin E (Cpd. **454**)) was carried out with the culture of the fungus *Chaetomium globosum* MP4-S01-7 from the deep sea. The elucidation of the structures of the new compounds was carried out by HSQC-HECADE NMR data, J-based configuration analysis, and modified Mosher’s method, and the verification was carried out by comparing the recorded and computed NMR chemical shifts from the quantum chemical calculations together with a DP4+ statistical procedure. The compounds were screened for an *in vitro* cytotoxic activity against the gastric cancer cell lines MGC803 and AGS. Most of the compounds inhibited cancer cell viability at about 10.0 μM, but Cpds. **446**, **447**, and **449** exhibited the most potent cytotoxic activity on the cancer cells, with IC_50_ values less than 1.0 μM (Table S3). Additionally, Cpd. **446** inhibited cell cycle progression, and both Cpds. **445** and **446** caused apoptosis in gastric cancer cells in a concentration-dependent manner [83]. The structures of Cpds. **445**, **446**, and **449** are in Figure 3.

A novel epidithiodiketopiperazine (DC1149Ba) (Cpd. **455**), isolated from *Trichoderma lixii,* an unidentified sponge from Mentawai, Indonesia, inhibited the proliferation of Panc-1 with an IC_50_ of 0.02 µM. This cell line was cultured in a glucose-deficient medium with a selectivity index of 35,500-fold higher for cells cultured under glucose-starved conditions than those under general culture conditions. Equally, this compound inhibited the response of the endoplasmic reticulum stress signaling, an effect which could be caused by inhibiting complex II in the mitochondrial electron transport chain [84]. The structure of epidithiodiketopiperazine (DC1149Ba) (Cpd. **455**) is in Figure 3.

From the Egyptian Red Sea marine sponge *Siphonochalina siphonella*, four new polyacetylene amides, siphonellamides A–D (Cpds. **456**–**459**), one new fatty amide, siphonellamide E (Cpd. **460**), a known indole fatty amide (Cpd. **461**), and callyspongamide A (Cpd. **462**) were isolated. The compounds were evaluated for their antiproliferative effect on the HeLa, MCF-7, and A549 malignant tumor cell lines. With the IC_50_ range of 9.4–34.1 μM, compounds **456** and **457** inhibited the proliferation of the cancer cells (Table S3) [85].

Bulbimidazoles A−C (Cpds. **463**−**465**) inhibited the growth of P388 murine leukemia cells with their IC_50_ in the micromolar range (Table S3). The compounds are new alkanoyl imidazoles that remained isolated from the culture extract of the *γ*-proteobacterium *Microbulbifer* sp. DC3-6 sourced from a stony coral of the genus Tubastraea. By using the Ohrui−Akasaka method, Cpd. **463** was determined to be a mixture of (R)- and (S)-configurations that occurred in a 9:91 ratio after the absolute configuration of the substituted anteiso-methyl was assigned [86].

Shellmycins A–D (Cpds. **466**–**469**) were the four novel cytotoxic tetrahydroanthra-γ-pyrone compounds isolated from *Streptomyces* sp. shell-016 from a marine shell sediment sample taken from Binzhou Shell Dike Island and Wetland National Nature Reserve, China. The compounds were antitumorigenic on the five cancer cell lines A375, H1299, HepG2, HT29, and HCC1937 that were screened, with IC_50_ values from 0.69–26.3 µM (Table S3). The putative biosynthetic pathways of the compounds were discussed based on their structure–activity relationship, and their structures were deduced by the interpretation of 1D and 2D NMR and HR-MS data [87]. The absolute configuration of Cpd. **466** was confirmed by single crystal X-ray diffraction, and Cpds. **468** and **469** are stereoisomers to each other. The structures of the compounds with cytotoxic IC_50_ ≤ 1.0 µM are in Figure 3 [87].

### 3.4. Marine-Derived Lipids, Sterols, and Steroids Cytotoxic to Cancer Cell Lines

Five new ergostanes, penicisteroids D−H (Cpds. **470**−**474**), and 27 known compounds (Cpds. **475**–**501**) were isolated from the liquid culture of the *Penicillium granulatum* MCCC 3A00475, a deep-sea-derived fungus. Some of the compounds exhibited moderate cytotoxic effects selectively on 12 cancer cell lines with IC_50_ values in the range of 5–16.6 µM (Table S4). Cpds. **471** and **475** were bound to the retinoid X receptors with Kd values of 13.8 and 12.9 µM, respectively, and could induce apoptosis by a retinoid X receptor (RXR)-αdependent regulation of RXRα transcriptional expression, thus, promoting poly-ADP-ribose polymerase (PARP) cleavage. In addition, they could be cytotoxic via cell cycle arrest at the G0/G1 phase [88].

The fermentation of the ethyl acetate extract of the deep-sea sediment fungus *Penicillum citreonigrum* XT20-134 (MCCC 3A00956) yielded twenty-four compounds of which five of them are new and include adeninylpyrenocine (Cpd. **502**), 2-hydroxyl-3-pyrenocine-thio propanoic acid (Cpd. **503**), ozazino-*cyclo*-(2,3-dihydroxyl-trp-tyr) (Cpd. **504**), 5,5-dichloro-1-(3,5-dimethoxyphenyl)-1,4-dihydroxypentan-2-one (Cpd. **505**), and 2,3,4-trihydroxybutyl cinnamate (Cpd. **506**). The remaining are known compounds (Cpds. **507**–**525**). All the isolates were tested for their antitumor activities against Bel7402 and HT1080 human cancer cells. It was only Cpds. **503** and **506** containing a heteroatom that inhibited the growth of those cancer cells with IC_50_ values of 7.63, 13.14 µM, and 10.22, 16.53 µM, respectively [89].

Two novel oxygenated ergostane-type sterols (Cpds. **526**, **527**) and one known related compound, sinugrandisterol A (Cpd. **528**), were isolated from the soft coral *Sinularia* sp. sourced from the Xisha Islands, South China Sea. In the antiproliferation screening, the new compounds inhibited the proliferation of MDA-MB-436, A549, Hep3B, HT-29, and H157 human cancer cell lines with moderate IC_50_ values (Table S4). Furthermore, the result revealed that Cpd. **528**-treated H157 cells, with Hoechst 33,258 staining, morphologically presented characteristics of apoptosis. In addition, the Western blot assay implies that Cpd. **528** could up-regulate and down-regulate the expressions of Bax and Bcl-2, respectively [90].

By following the one-strain-many-compounds approach, seven culture media of *Clonostachys rosea* MMS1090 were optimized to increase the yield of eburicol (Cpd. **529**). After the purification and structural analysis by NMR, eburicol was tested against four human cancer cell lines, MCF-7, MDA-MB-231, NSCLC-N6-L16, and A549. However, eburicol could inhibit the growth of MCF-7 and MDA-MB-231 cells with 2.0 and 15.7 µM IC_50_ values, respectively. This is the first time the accumulation of eburicol in the marine fungal strain *C. rosea* has been reported, and this confirms its potential to produce bioactive lipids [91].

From the coasts of Dokdo Island in the Republic of Korea, the marine sediment actinomycete *Actinoalloteichus hymeniacidonis* was collected. This fungus yielded three new hydroxylated rhamnolipids, dokdolipids A, B, and, C (Cpds. **530**–**532**). The three compounds were evaluated for cytotoxic activity against six human cancer cell lines, HCT-15, NUGC-3, NCI-H23, ACHN, PC-3, and MDA-MB-231. Cpds. **530**–**532** were moderately cytotoxic to the cell lines with IC_50_ values in the range of 13.7–41.5 µM (Table S4) [92].

### 3.5. Marine-Derived Ketones, Quinines, Quinolones, and Xanthones Cytotoxic to Cancer Cell Lines

Two new medermycin derivatives, lactoquinomycin C (Cpd. **533**) and lactoquinomycin D (Cpd. **534**), along with six known compounds (Cpds. **535**, **536**, **537**, **538**, **539**, and **540**), were obtained from rice-solid fermentation of *Streptomyces* sp. SS17A isolated from the marine environment. Lactoquinomycin D (Cpd. **535**) contains 5, 14-epoxidation which is a rare structural feature in medermycin derivatives. Cpds. **533** and **534** were not cytotoxic to PC-3 and HCT-116 human malignant tumor cell lines, but 3-methyl-antibiotic G-15F (Cpd. **535)** inhibited their growth strongly with IC_50_ values of 0.02 and 0.04 µM, respectively (Table S5). From the SAR study, the γ-lactone unit is necessary for significant cytotoxicity regarding Cpds. **533**–**535** [93].

Two new dimeric 1, 4-benzoquinone derivatives, peniquinone A (Cpd. **541**) and peniquinone B (Cpd. **542**), a new dibenzofuran penizofuran A (Cpd. **543**), and a new pyrazinoquinazoline derivative quinadoline D (Cpd. **544**), along with 13 known compounds (Cpd. **545**–**547**), were isolated from the marine-derived fungus *Penicillium* sp. L129. With the following respective IC_50_ (µM) values, 12.39, 9.01, and 14.59, Cpd. **541** inhibited the proliferation of MCF-7, U87, and PC3 human neoplasms, but Cpd. **543** inhibited their growth with higher IC_50_ values of 25.32, 13.45, and 19.93 µM, respectively [94].

The novel lithocarols A–F (Cpds. **558**–**563**) that possessed a highly oxygenated isobenzofuran core, along with a related known isoprenylisobenzofuran A (Cpd. **564**), were isolated from *Phomopsis lithocarpus* FS508, a marine-derived fungus. Lithocarols A–E (Cpds. **558**–**562**) were the first poly-ketal derivatives in the tenellone family. Cpds. **558**–**561** were moderately cytotoxic to four human malignant tumor cell lines with the IC_50_ range of 10.5–38.7 μM (Table S5) [95].

The methanol extract of the Vietnamese octocoral *Dendronephthya mucronata* yielded four bicyclic lactones A–D (Cpds. **565**, **566**, **567**, and **568**), which includes three new compounds named dendronephthyones A–C (Cpds. **565, 566,** and **567**) after a series of chromatographic purifications. From the bioassay screening, Cpds. **565**–**567** exhibited weak selective cytotoxic activity against the HeLa human malignant tumor cell line with IC_50_ values of 32.48, 30.12, 35.14, and 14.45 µM (Table S5), respectively, but not against A-549 and Panc-1 (IC_50_ > 100 µM) [96].

The co-culture of *Aspergillus versicolor* (a sponge-derived fungus) and *Bacillus subtilis* on rice-solid media resulted in the isolation of the following compounds: a new cyclic pentapeptide (cotteslosin C, Cpd. **569**), a new aflaquinolone (22-epi-aflaquinolone B, Cpd. **570**), two new anthraquinones (Cpds. **571** and **572**), and 30 known Cpds. (**573**–**602**). The new analogues were obtained only in the co-culture extract, and not in the axenic fungal culture. In addition, the co-culture extract increased the buildup of the known compounds versicolorin B (Cpd. **574**), averufin (Cpd. **584**), and sterigmatocyctin (Cpd. **587**) by factors of 1.5, 2.0, and 4.7, respectively, in comparison to the axenic fungal culture. Out of these isolated compounds, only five of them (O-demethylsterigmatocystin (Cpd. **586**), sterigmatocystin (Cpd. **587**), sterigmatin (Cpd. **588**), AGI-B4 (Cpd. **589**), and stephacidin A (Cpd. **594**)), moderately inhibited the growth of mouse lymphoma cell line L5178Y, with the IC_50_ range of 2.0–21.2 µM (Table S5) [97]. 

The bioactivity-guided purification of the extract from *Streptomyces bingchenggensis* ULS14 from Lagos Lagoon in Nigeria gave two known compounds, ULDF4 (kigamicin) (Cpd. **603**) and ULDF5 (staurosporine) (Cpd. **604**). The IC_50_ values of ULDF4 and ULDF5 against the proliferation of the human cancer cell line, HeLa were 0.11 and 0.24 µM/mL, respectively. This finding was the first report of the anticancer potential of actinomycetes from Lagos Lagoon, which can be used for potential drug developmental purposes [98].

### 3.6. Other Marine-Derived Compounds Cytotoxic to Cancer Cell Lines

**Figure 4:**
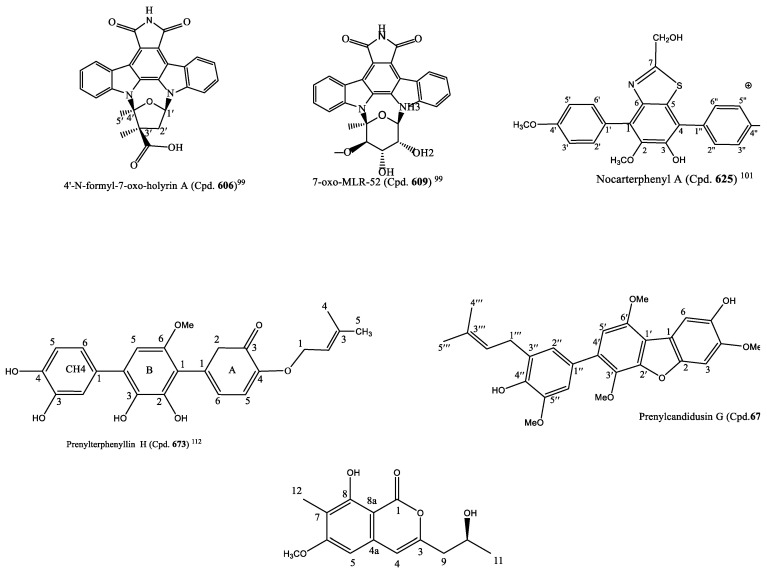
The structure of other novel marine-derived compounds cytotoxic to cancer cell lines at IC_50_ ≤ 1 µM.

The rice-solid fermentation of the marine-derived *Streptomyces* sp. NB-A13 produced six new (Cpd. **605**–**610**) and nine known (Cpd. **611**–**619**) staurosporine derivatives. The compounds were screened for anticancer activity against PC-3 and SW-620 cell lines. With the IC_50_ value of 0.01 µM, Cpd. **611** was the most potent and inhibited the proliferation of SW-620 cell line more than the staurosporine positive control (Cpd. 18) (0.025 µM) and Cpds. **605**–**609**, **612**–**617**, and **619** that had IC_50_ values ranging from 0.02 to 16.60 μM; Cpd. **610** exhibited no cytotoxic potency (Table S6). All the compounds were more selective for SW-620 than the PC-3 cells, although they had a similar potency on both cancer cell lines (Table S6). A structure–activity relationship of the derivatives revealed a bisindolocarbazole, and the bridged sugar units are necessary for the cytotoxicity based on the fact that compounds **611** and **618** are the most active as well as Cpds. **609**, **614**, and **617** compared to the other analogues. Cpd. **611** was more potent than Cpd. **618** that served as a positive control. Additionally, Cpd. **611** more strongly inhibited the activities of protein kinase C-theta (PKC-θ) at IC_50_ of 0.06 µM than the other analogues (Cpds. **605**–**610** with a range of IC_50_ of 0.06-9.43 μM) except for Cpd. **610** with no inhibitory activity, but it was close to that of the positive control (staurosporine (Cpd.14; IC_50_ = 0.01 µM)). These findings suggest that the discovered staurosporine derivatives with considerable cytotoxic and PKC-θ inhibitory activities could be important materials for the development of new anticancer agents [99]. The structures of Cpds. **606** and **609** are in Figure 4.

The isolation of the five new decalin derivatives, altercrasins A, B, C, D, E (Cpds. **620**–**624**), was carried out with *Alternaria* sp. OUPS-117D-1; a strain initially derived from *Anthocidaris crassispina*, a sea urchin. The absolute stereo-structure of altercrasin A (Cpd. **620**) was determined by chemical transformation and the modified Mosher’s method. Silica gel and reversed-phase high-performance liquid chromatography (RP-HPLC) were used to purify altercrasins B, C, D, and E (Cpds. **621**–**624**), and their structures were elucidated by using 1D and 2D nuclear magnetic resonance (NMR) spectroscopic analyses. By comparing the NMR chemical shifts, NOESY correlations, and electronic circular dichroism (ECD) spectral analyses of Cpd. **620**, the absolute configurations of Cpds **621**–**624** were deduced. Subsequently, the authors discovered that Cpd. **620** was the stereoisomer of Cpd. **621** and Cpd. **623** was a stereoisomer of Cpd. **624**. Additionally, their cytotoxic activities were screened against the murine P388 leukemia, human HL-60 leukemia, and murine L1210 leukemia cell lines; the result revealed that Cpds. **623** and **624** exhibited a potent cytotoxic effect on the cancer cells (Table S6) [100].

The marine-derived actinobacterial strain *Nocardiopsis* sp. OUCMDZ-4936 produced three new p-terphenyl derivatives, nocarterphenyls A-C (Cpds. **625**–**627**) and three known analogues (Cpds. **628**–**630**). Cpd. **625** possessed a benzothiazole and Cpd. **627** a benzothiazine moiety. These moieties are not common in the skeleton of p-terphenyls. The compounds exhibited potent cytotoxic activity against some of the cell lines among the 26 human cancer cell lines used for the screening, with the range of 0.10–17.0 μM IC_50_ values (Table S6) [101]. The structure of nocartephenyl A is in Figure 4.

The purification of the fermented broth of *Streptomyces* sp. RKND004, sourced from Prince Edward Island sediment, yielded two new polycyclic polyether compounds, terrosamycins A (Cpd. **631**) and B (Cpd. **632**). The compounds were identified by the OSMAC approach and a UPLC-HRMS-based metabolomics assay. By using NMR, HRMS, and X-ray diffraction data analyses, the structure of Cpd. **631** was deduced. From the spectral data analysis of Cpd. **632**, there are attached two methoxy groups that replaced the two hydroxy groups in Cpd. **631**; that implies the methoxy group is required for the anticancer activity. Cpd. **631** preferentially binds potassium over sodium. Like other polyether ionophores, Cpds. **631** and **632** inhibited the growth of two breast cancer cell lines with moderate IC_50_ values in the range of 3.6–10.1 µM (Table S6). Moreover, these compounds were more selective for the cancer cells than the normal cells (human foreskin fibroblast cell line (BJ) (IC_50_ = 79 and 267 µM, respectively) and healthy Cercopithecus aethiops kidney epithelial cells (Vero) (IC_50_ = 36 and 93 µM, respectively)) that were screened too [102]. Thus, it is worth mentioning that the terrosamycins were more potent than salinomycin that has progressed to pilot clinical trials for the treatment of breast cancer and other cancers.

The previously isolated oxadiazine, nocuolin A (NocA) (Cpd. **633**) from the cyanobacterial strain *Nodularia* sp. LEGE 06071, was screened for anticancer activity against a colon cancer cell line (HCT116) and an immortalized epithelial cell line (hTERT RPE-1). The cytotoxic efficacy of NocA was significant in the cancer cells undergoing exponential growth but was not for non-proliferating immortalized cells [103]. It induced apoptosis in HCT116 multi-cellular spheroids by suppressing the overexpression of the *bcl* genes. Amazingly, the result of the transcriptome analysis of the HCT116 cells did not relate to any compound in the CMap. However, it pointed to stress responses and cell starvation, which may be due to a decrease in adenosine triphosphate (ATP) production. Additionally, autophagy and a decrease in the mitochondrial respiration parameter within one hour of treatment were recorded. A similar study also concluded that nutritionally starved spheroid cells are sensitive to impaired mitochondrial energy production due to limited metabolic plasticity, therefore, suggesting NocA as a mitochondrial toxin to HCT-116 [104].

The following ten new di-, tri-, and tetrasulfated triterpene glycosides, psolusoside B1 (Cpd. **634**), B2 (Cpd. **635**), J (Cpd. **636**), K (Cpd. **637**), L (Cpd. **638**, M (Cpd. **639**), N (Cpd. **640**), O (Cpd. **641**), P (Cpd. **642**), and Q (Cpd. **643**), were isolated from *Psolus fabricii*, a sea cucumber from Okhotsk, close to the Kurile Islands. The structures of these compounds were deduced by two-dimensional (2D) NMR spectroscopy and HR-ESI mass spectrometry. Cpds. **642** and **643** are highly polar and have four sulfate groups in their carbohydrate moieties, plus two sulfates in the same terminal glucose residue. Psolusoside B2 (Cpd. **636**) has an atypical non-holostane aglycone with 18 (16)-lactone and a distinctive 7, 8-epoxy fragment. The cytotoxicity screening of the compounds against the following mouse cancer cell lines, Ehrlich ascites carcinoma, neuroblastoma Neuro 2-A, and erythrocytes, was carried out. Psolusoside L (Cpd. **638**) (a pentaoside) that has three sulfate groups at C-6 of two glucoses and one 3-*O*-methylglucose residue and holostane aglycone, was the most active compound among the others (Table S6). The structure–activity relationship revealed that the sulfate group at C-2 of the terminal glucose residue attached to C-4 of the first xylose residue extensively reduced activities of the corresponding glycosides. Psolusosides of group B1 (Cpd. **634**), B2 (Cpd. **635**)**,** and the known psolusoside B) did not show any activity in any of the screenings because of the attachment of non-holostane aglycones and tetrasaccharide-branched sugar chains to the sulfate group of carbon-2 of psolusoside K (Cpd. **637**) [105].

One novel (Cpd. **644**) and three known (Cpds. **645**–**647**) p-terphenyl derivatives were isolated from the deep-sea-derived fungus *Aspergillus candidus*. The compounds were tested for in vitro antifood allergic and anticancer activities. Cpds. **646** and **647** exhibited potent anticancer activity against four human cancer cell lines, HeLa, Eca-109, Bel-7402, and Panc-1, with the IC_50_ range of 5.5 to 9.4 l µM (Table S6) [106].

By using the one-strain-many-compound (OSMAC) technique on *Aspergillus* sp. LS34, a sponge-derived fungus, cultured in two different media (a solid-rice medium and potato dextrose broth (PDB)), two new compounds, asperspin A (Cpd. **648**) and asperther A (Cpd. **649**), plus seven known Cpds. **650**–**656**, were isolated. The fungal extracts cultured in the rice medium produced Cpds. **648**–**652** and in the PDA medium, Cpds. **653**–**656**. From the anticancer activity study carried out on the compounds against the seven cancer cell lines, CCRF-CEM, K562, BGC823, AGS, HCT-116, MDA-MB-453, and COR-L23, only Cpd. **656** had significant antiproliferative activity on CCRF-CEM and K562 cells with 1.22 and 10.58 µM IC_50_ values, respectively (Table S6) [107].

By the use of 1D, 2D NMR, and HR-ESI-MS spectroscopic techniques, the structures of one new compound, named holothurin A5 (**Cpd. 657**), and eight known triterpene glycosides (Cpds. **658**–**665**), that were obtained from the methanol extract of the Vietnamese sea cucumber *Holothuria edulis*, were deduced. Holothurin A5 (Cpd. **657**) has a hydroperoxy group at C-25. That was the first report of the isolation of this group of triterpene saponin compounds from sea cucumbers [108]. In addition, the *in vitro* cytotoxicity of the compounds was screened against five human cancer cell lines (HepG2, KB, LNCaP, MCF7, and SK-Mel-2). The compounds, especially Cpds. **659, 663,** and **664**, had mild to moderate to strong cytotoxic activity on the cancer cells (Table S6) [109].

A dark-red wooly textured *Cyanobacterium* was collected from the marine environment in Boca del Drago, Panama. It produced four new dragocins A-D (Cpds. **666–669**) after extraction with a 2:1 ratio of a dichloromethane:methanol mixture and further chemical treatments. Dragocins A-C (Cpds. **666**–**668**) have 2, 3-dihydroxypyrrolidine, 1-hydroxy-5-O-Me-benzoyl, and 4′-substituted β-ribofuranose moieties that were fused to form a nine-membered macrocyclic ring. Dragocins A, B, and C (Cpds. **666**–**668**) were characterized by substitution at the C-4′ position of a ribofuranose unit. Moreover, with the IC_50_ of 7.6 µM, dragocin A was cytotoxic to human H-460 lung cancer cells (Table S6) [110].

Asfour and co-authors succeeded in the large-scale production of pure terrein (Cpd. **670**), a compound obtained from *Aspergillus terreus* strain S020, after culturing in a 21 L fermentation static broth for 40 days at room temperature on potato dextrose broth and a series of chemical treatments and chromatographic separations, in a yield of 537.26 ± 23.42 g/kg extract; thus, this represents the highest yield of terrein produced by fermentation to date. After the cytotoxic bioactivity-guided screening of the fungal extracts, the isolated compound terrein was also screened against human colorectal (HCT-116) and hepatocellular (HepG2) carcinoma cell lines. It exhibited cytotoxicity on the cells with IC_50_ values of 12.13 and 22.53 µM (Table S6), respectively. This study, therefore, suggests *A. terreus* strain S020 as a good source for the large-scale production of terrein in industries [111].

The following nine new prenylated p-terphenyls, prenylterphenyllins F-J (Cpds. **671**, **672**, **673**, **674**, **675**) and prenylcandidusin D-G (Cpds. **676**, **677, 678**, **679**), were sourced from an endophytic fungus, *Aspergillus candidus* LDJ-5. Their structures were determined from NMR and MS data. In contrast to a previously reported study on p-terphenyls, Cpd. **676** has a rare 6, 5, 6, 6-fused ring system. With this range (0.40–16.9 µM) of IC_50_ values, Cpds. **681**, **682**, **675**, and **679** were cytotoxic to a panel of nine human cancer cell lines (Table S6) [112]. The structure of prenylterphenyllin H (Cpd. **673**) is in Figure 4 [112].

The following three new monarubins, A, B, and C (Cpds. **680**, **681**, and **682**), and ten known compounds, four alkaloids (Cpds. **683**–**686)**, two isocoumarins (Cpds. **687** and **688**), and four polyketides (Cpds. **689**–**692**), were isolated from a marine shellfish-associated fungus *Monascus ruber* BB5; they were screened for antiproliferative activity against human nasopharyngeal (CNE1, CNE2, CNE1, and HONE1) and hepatocellular (QGY7701 and HepG2) carcinoma cell lines. Monarubin B (Cpd. **680**) was potently cytotoxic to HepG2 and QGY7701 with the respective IC_50_ values of 1.72 and 0.71 μΜ; lunatinin (Cpd. **686**) was moderately cytotoxic to HepG2, QGY770, and SUNE1 with the respective IC_50_ values of 9.60, 7.12, and 28.12 μΜ. [113].

Meleagrin (novel Cpd. **693**) and other known compounds, haenamindole (Cpd. **694**), isorugulosuvine (Cpd. **695**), secalonic acid D (Cpd. **696**), ergosterol (Cpd. **697**), and cerebroside A (Cpd. **698**), were isolated from the fungus *Emericella dentata* Nq45. From the result of the cytotoxicity screening of maleagrin (Cpd. **693)** against the human cervix carcinoma cell line KB-3-1 and its multi-drug-resistant sub-clone KB-V1, meleagrin (Cpd. **693)** was cytotoxic to cells with IC_50_ values of 3.07 and 6.07 µM, respectively (Table S6) [114].

Guo et al. investigated the effect of culturing the marine-derived fungus *Trichoderma erinaceum* F1-1 in glucose–peptone–yeast (GPY) medium supplemented with a single amino acid, L-phenylalanine, or not. Seven terpenoids (Cpds. **699**–**705**) and one polyketide (Cpd. **706**), out of which four are new compounds, namely, harziandione A (Cpd. **699**), cyclonerodiols A (Cpd. **701**) and B (Cpd. **702**), and trichodermaerin A (Cpd. **704**), from the GPY medium without L-phenylalanine, and 18 aromatic compounds (Cpds. **707**–**722**), which include six new compounds, named trichoderolides A–F (Cpds. **707**, **708**, and **712**–**715**), from the GPY medium with L-phenylalanine were obtained. Cpd. **708**, **710**, and **711** were cytotoxic to a human melanocyte cancer cell-MDA-MB-435. Cpd. **724** was cytotoxic to A549 human alveolar basal epithelial cell adenocarcinoma (Table S6) [115].

By searching for new anticancer agents from marine samples, Fan et al. isolated these compounds: three new decalinoylspirotetramic acid derivatives, pyrenosetins A, B, and C (Cpds. **725**–**727**), and one known decalin tetramic acid, phomasetin (Cpd. **728**), from the C18-SPE fractions of the trichloromethane sub-extract of an endophyte *Pyrenochaetopsis* sp. FVE-001, a fungus derived from the brown alga *Fucus vesiculosus.* Cpds. **725** and **726** exhibited antitumor activity against the melanoma neoplasm cell line A-375 with IC_50_ values of 2.8 and 6.3 µM (Table S6), respectively. That is the first report of isolated secondary metabolites from the marine-derived *Pyrenochaetopsis* sp. and the second from the genus *Pyrenochaetopsis* [116].

From marine sediment in a volcanic island in Korea, *Streptomyces* sp. strain SUD1 was isolated. The chemical profiling of the bacteria led to the isolation of the three new metabolites, donghaecyclinones A–C (Cpds. **729**–**731**). Donghaecyclinones A–C (Cpds. **729**–**731**) demonstrated cytotoxic activity against diverse human cancer cell lines (IC_50_: 6.7–9.6 μM for Cpd. **731**) (Table S6) [117].

## 4. Conclusions

There has been a growing rate of cancer incidence worldwide due to factors such as an aging population, eating habits, environmental changes, and co-morbidities. Poor treatment outcomes and complications of cancer disease are aggravated by multi-drug resistant malignant tumors, the high cost of treatment, and adverse drug reactions/patient compliance. Therefore, there is high demand for new anticancer agents or for modifying the existing ones. This necessitated the search for potent and efficacious substances from marine sources that will improve or replace existing ones in order to contain multi-drug-resistant neoplasms. This review compiled a total of 731 compounds/derivatives that belong to these classes: glycosides, alkaloids, saponins, lipids, terpenes, ribose, steroids, peptides, xanthones, ethers, lignins, coumarins, carbazoles, azaphilones, nucleosides, polyketides, and quinones (Tables S1–S6). These compounds/derivatives sourced from soft corals, bacteria, fungi, sponges, algae, sea cucumbers, seaweeds, mollusks, and sea urchins exhibited moderate to high cytotoxic activities against 121 mammalian cancer cell lines as described in 76 articles from January 2019–March 2020. The IC_50_ (≤20.0 µM) values of both known and novel compounds were shown with structures of the new compounds. Interestingly, some of these compounds exhibited a broad spectrum of anticancer activity and are worth further exploration in terms of mechanism of action, structure–activity relationship, and clinical trials. However, this could serve as a guide for the selection of novel compounds/derivatives for future optimization and drug development.

## Data Availability

Not applicable.

## Supplementary Materials

The following are available online at https://www.mdpi.com/1420-3049/26/19/5769/s1. Table S1: The IC50 of marine derived alkaloids with anticancer activity, Table S2: The IC50 of marine–derived terpenes and terpenoids with anticancer activity, Table S3: The IC50 of marine-derived amino acids, peptides, and polyketides with anticancer activity, Table S4: The IC50 of marine-derived lipids, sterols and steroids with anticancer activity, Table S5: The IC50 of marine-derived ketones, quinines, quinolones and xanthones with anticancer activity, Table S6: The IC50 of other marine-derived compounds with anticancer activity.
