# Supplementary material for: Novel Marine Secondary Metabolites Worthy of Development as Anticancer Agents: A Review
Source: Molecules. 2021 Sep 23;26(19):5769. doi: 10.3390/molecules26195769 (PMC8510081; doi:10.3390/molecules26195769)
Supplement: Supplementary file 1 [file molecules-26-05769-s001.zip › molecules-1355438-supplementary.pdf]

molecules

MDPI

Supplementary Materials
Novel Marine Secondary Metabolites Worthy of Development as Anticancer Agents: A Review

Florence Nwakaego Mbaoji [1,2,3], Justus Amuche Nweze [1,4,5,6], Liyan Yang [7], Yangbin Huang [1], Shushi Huang [1], Akachukwu Marytheresa Onwuka [3], Ikechukwu Emmanuel Peter [3], Cynthia Chioma Mbaoji [3], Mingguo Jiang [8], Yunkai Zhang [2,*], Lixia Pan [7,*] and Dengfeng Yang [1,*]

1 Guangxi Key Laboratory of Marine Natural Products and Combinatorial Biosynthesis Chemistry, Guangxi Beibu Gulf Marine Research Center, Guangxi Academy of Sciences, Nanning 530007, China;
florence.mbaoji@unn.edu.ng (F.N.M.); justus.nweze@unn.edu.ng (J.A.N.);
yangbinhuang106@163.com (Y. H.); hshushi@gxas.cn (S.H.)

2 College of Life Science and Technology of Guangxi University, Nanning 530004, China

3 Department of Pharmacology and Toxicology, Faculty of Pharmaceutical Sciences, University of Nigeria, Nsukka 410001, Enugu State, Nigeria;
akachukwu.onwuka@unn.edu.ng (A.M.O.);
ikechukwu.peter@unn.edu.ng (I.E.P.); cynthia.mbaoji.194479@unn.edu.ng (C.C.M.)

4 Department of Science Laboratory Technology, Faculty of Physical Sciences, University of Nigeria, Nsukka 410001, Enugu State, Nigeria

5 Department of Ecosystem Biology, Faculty of Science, University of South Bohemia in Ceske Budejovice, 37001 Ceske Budejovice, Czech Republic

6 Soil and Water Research Infrastructure, Biology Centre, Czech Academy of Sciences, 10000 Prague, Czech Republic

7 Guangxi Biomass Industrialization Engineering Institute, National Engineering Research Center of Non-food Biorefinery, State Key Laboratory of Non-Food Biomass, Guangxi Academy of Sciences, Nanning 530007, China; yangliyan.1988@163.com

8 Guangxi Key Laboratory for Polysaccharide Materials and Modifications, School of Marine Sciences and Biotechnology, Guangxi University for Nationalities, Nanning 530008, China; mzxyjiang@163.com

* Correspondence: yykzhang@gxu.edu.cn (Y.Z.); panlixia10@163.com (L.P.); dengfengyang@163.com (D.Y.); Tel: +86-771-2503980 (L.P.); +86-771-2536109 (D.Y.)

molecules

MDPI

Table of Contents

S/N	Title	Page
S1	The IC_{50} of marine derived alkaloids with anticancer activity	3
S2	The IC_{50} of marine–derived terpenes and terpenoids with anticancer activity	8
S3	The IC_{50} of marine-derived amino acids, peptides, and polyketides with anticancer activity	13
S4	The IC_{50} of marine-derived lipids, sterols and steroids with anticancer activity	19
S5	The IC_{50} of marine-derived ketones, quinines, quinolones and xanthones with anticancer activity	21
S6	The IC_{50} of other marine-derived compounds with anticancer activity	22

molecules

MDPI

Table S1. The IC_{50} of marine derived alkaloids with anticancer activity

S/N	Isolated Compounds	Class	Status	Source	Year	Cancer cell line	Cancer Source	IC_{50} (µM)	Ref.
1.	Subereamolline D (Cpd. **1**)	Bromotyrosine-derived alkaloids	Novel	Sea sponge *Fascaplysinopis reticulata* from Xisha Islands in the south China	2019	Jurkat cell (*T-cell leukaemia*)	Human	0.88	37
2.	N-glutarylchaetoviridin A (Cpd. **2**)	Glutamine-containing Azaphilone Alkaloids	Novel (Cpds. **2**, **4**)	Deep-Sea-Derived Fungus *Chaetomium globosum* HDN151398, a fungus isolated from a deep-sea sediment sample collected in South China Sea.	2019	HL-60 (*leukaemia*)	All human	10.3	38
	N-glutarylchaetoviridin *C* (Cpd. **4**)					HL-60 (*leukaemia*)<br>K562 *rhabdomyoma*)<br>BEL-7402 (*hepatoma*)<br>HCT-116 (*colon*)<br>L-02 (*hepatic*)<br>MGC-803 (*gastric*)<br>HO8910 (*ovary*)<br>(*neuroblastoma*)<br>NClH1975 (*lung adenocarcinoma*)<br>U87 (*glioblastoma*)<br>MDA-MB-231(*breast*)		11.1<br>11.7<br>10.9<br>11.3<br>18.2<br>6.6<br>9.7<br>11.2<br>18.3<br>13.2	
	Chaetomugilin A (Cpd. **5**)		Known (Cpds. **5**, **6**)			HL-60 (*lukaemia*)<br>K562 *rhabdomyoma*)<br>BEL-7402 (*hepatoma*)<br>HCT-116 (*colon*)<br>L-02 (*hepatic*)<br>MGC-803 (*gastric*)<br>HO8910 (*ovary*)		6.4<br>11.1<br>17.9<br>6.1<br>15.2<br>15.3<br>12.1<br>18.3	

						NClH1975 (*lung*)		6.6	
	Chaetomugilin C (Cpd. **6**)					HL-60 (*leukaemia*)<br>K562 (*rhabdomyoma*)<br>BEL-7402 (*hepatoma*)<br>HCT-116 (*colon*)<br>HeLa (*cervical*)<br>L-02 (*hepatic*)<br>MGC-803 (*gastric*)<br>HO8910 (*ovary*)<br>SHSY5Y<br>(*neuroblastoma*)<br>NClH1975 (*lung*)<br>U87 (*glioblastoma*)		12.3<br>16.8<br>5.7<br>13.2<br>9.1<br>9.6<br>8.8<br>19.4<br><br>12.1<br>17.6	
3.	Monanchoxymycalin C (Cpd. **12**)	Pentacyclic guanidine alkaloid	Novel	Marine sponge *Monanchora pulchra* collected off the coast of Chirpoi Island	2019	HELa (*cervical*)	Human	3.5	39
4.	Araguspongine C (Cpd. **14**)	Macrocyclic bis-quinolizidine alkaloid	Novel (Cpd. **15**) others known	Marine sponge *Xestospongia muta* collected at the coral reef of Vinh Moc, Quang Tri, Vietnam	2019	HepG-2 (*liver*)<br>HL-60 (*leukaemia*)<br>LU-1 (*lung*)<br>MCF-7 (*breast*)<br>SK-MEL-2 (*Skin*)	All human	0.75<br>0.88<br>0.96<br>0.79<br>1.02	40
	Meso-araguspongine C (Cpd. **15**)					HepG-2 (*liver*)<br>HL-60 (*leukaemia*)<br>LU-1 (*lung*)<br>MCF-7 (*breast*)<br>SK-MEL-2 (*Skin*		0.43<br>0.62<br>0.76<br>0.44<br>0.77	
	Araguspongine N (Cpd. **18**)					HepG-2 (*liver*)<br>HL-60 (*leukaemia*)<br>LU-1 (*lung*)<br>MCF-7 (*breast*)<br>SK-MEL-2 (*Skin*		6.58<br>7.84<br>9.20<br>7.36<br>11.23	
	Araguspongines O (Cpd. **19**)					HepG-2 (*liver*)<br>HL-60 (*leukaemia*)<br>LU-1 (*lung*)<br>MCF-7 (*breast*)<br>SK-MEL-2 (*Skin*		5.06<br>5.65<br>5.63<br>5.32<br>5.45	
	Araguspongine P (Cpd. **20**)					HepG-2 (*liver*)<br>HL-60 (*leukaemia*)<br>LU-1 (*lung*)<br>MCF-7 (*breast*)<br>SK-MEL-2 (*Skin*		5.55<br>6.58<br>5.84<br>5.68<br>6.24	
	Araguspongine A (Cpd. **13**)					HepG-2 (*liver*)<br>HL-60 (*leukaemia*)<br>LU-1 (*lung*)		6.85<br>9.19<br>9.88	

Molecules **2021**, *26*, 5769. https://doi.org/10.3390/molecules26195769 www.mdpi.com/journal/molecules

						MCF-7 (*breast*)<br>SK-MEL-2 (*Skin*		7.82<br>7.51	
5.	Ascomylactam A (Cpd. **23**)	Macrocyclic alkaloids	All novel	Fungus CYSK-4 isolated from a marine semi mangrove Pluchea indica collected from Shankou Mangrove Nature Reserve in Guangxi Province, China	2019	MDA-MB-435 (*melanoma*)<br>MDA-MB-231 (*breast*)<br>SNB19 (*glioblastoma*)<br>HCT116 (*colon*)<br>NCI-H460 (*lung*)<br>PC-3 (*prostate*)	All human	4.9<br>5.9<br>6.8<br>5.5<br>4.4<br>5.7	41
	Ascomylactam B (Cpd. **24**)					MDA-MB-435 (*melanoma*)<br>MDA-MB-231 (*breast*)<br>SNB19 (*glioblastoma*)<br>HCT116 (*colon*)<br>NCI-H460 (*lung*)<br>PC-3 (*prostate*)		12<br>6.6<br>18<br>4.5<br>13<br>20	
	Ascomylactam C (Cpd. **25**)					MDA-MB-435 (*melanoma*)<br>MDA-MB-231 (*breast*)<br>SNB19 (*glioblastoma*)<br>HCT116 (*colon*)<br>NCI-H460 (*lung*)<br>PC-3 (*prostate*)		7.8<br>5.1<br>7.8<br>4.2<br>4.4<br>7.5	
6.	Chaetoglobosins E (Cpd. **33**)	Alkaloids	Known	*Chaetomium globosum* C2F17 isolated from a coral *Pocillopora damicornis* collected from the seashore near Sanya Bay, Hainan Province, China	2020	K562 (*chronic myelogenous leukaemia*)<br>A549 (*lung adenocarcinoma*)<br>Huh7 (*hepatocarcinoma*)<br>H1975 (*lung adenocarcinoma)*<br>MCF-7 (*breast carcimoma*)<br>U937(*lymphocytic leukemia*)<br>BGC823 (*gastric adenocarcinoma*)<br>HL60 (*promyelocytic leukemia*)<br>Hela (*cervical carcinoma*)	All human	8.9<br>5.9<br>1.4<br>9.2<br>2.1<br>1.4<br>8.2<br>2.5<br>2.8	42

	Fex (Cpd. **38**)					MOLT-4 (*acute T lymphoblastic leukaemia*)<br>Huh7 (*hepatocarcinoma*)<br>MCF-7 (*breast carcimoma*)<br>U937(*lymphocytic leukemia*)<br>MOLT-4 (*acute T lymphoblastic leukaemia*)		1.4<br>3.0<br>7.5<br>4.9<br>2.9	
7.	Tridiscorhabdin (Cpd. **45**)	Alkaloid	Novel	Sponge *Latrunculia biformis* Collected from southern Weddell deep sea (Antarctica)	2020	HCT-116 (*colon*)	Human	0.31	43
8.	7'-demethoxyrostratin C (Cpd. **50**)<br>Rostratin C (Cpd. **57**)	Thiodiketopiperazine alkaloids	Novel<br>Known	Fungal strain *Epicoccum nigrum* SD-388 was isolated from the deep-sea sediment collected in the West Pacific	2020	Huh7.5 (*Liver cancer*)	Human	9.52<br>4.88	44
9.	Streptoglutarimide H (Cpd. **65**)<br>Streptovitacin A (Cpd. **68**)	Glutarimide (Alkaloids)	Novel<br>Known	Marine-derived actinomycete, Streptomyces sp. ZZ741 from the coastal area of Jintang Island in Zhoushan, China	2019	U87MG *(glioma)*<br>U251*(glioma)*<br>U87MG *(glioma)*<br>U251*(glioma)*	All Human	3.8<br>1.5<br>0.22<br>0.05	45
10.	N-(3-Hydroxyphenethyl)-1-(1H-indole-3-carbonyl)-9Hpyrido[3,4-b]indole-3-carboxamide, 9e (Cpd. **73**)<br>N-(4-hydroxy-3-methoxybenzyl)-1-(1H-indole-3-carbonyl)-9H-pyrido[3,4-b]indole-3-carboxamid, 9l (Cpd. **80**)<br>Pityriacitrin - 1-(1H-indole-3-carbonyl)eN-(2-(methylsulfonyl)ethyl)-9Hpyrido[3,4-b]indole-3-carboxamide 9o (Cpd. **83**)<br>Methyl 2-(1-(1H-indole-3-carbonyl)-9H-pyrido[3,4-b] indole-3-carboxamido)-3-hydroxypropanoate 9p (Cpd. **84**)	β- carboline derivative of Pityriacitrin Indole Alkaloid	All novel	Marine *Burkholderia* sp. NBF227 from China	2019	SGC-7901 (*gastric*)<br>A875 (*melanoma*)<br>HepG2 (*liver*)<br>MARC145 (*Kidney*)<br>SGC-7901 (*gastric*)<br>A875 (*melanoma*)<br>HepG2 (*liver*)<br>MARC145 (*Kidney*)<br>SGC-7901 (*gastric*)<br>A875 (*melanoma*)<br>HepG2 (*liver*)<br>MARC145 (*Kidney*)<br>SGC-7901 (*gastric*)<br>A875 (*melanoma*)<br>HepG2 (*liver*)	Human<br>Human<br>Human<br>African green monkey	17.65<br>11.91<br>8.63<br>13.77<br>16.18<br>8.47<br>11.08<br>6.80<br>8.43<br>7.69<br>7.19<br>14.30<br>10.46<br>9.99<br>16.27	46
11.	Ochrazepine A (Cpd. **85**)	Circumdatins derivatives alkaloid	Novel (Cpds. **84**-**87**)<br>Known (Cpds. **88**,**89**)	Fungus *Aspergillus ochraceus* LCJ11-102 isolated from the coral *Dichotella gemmacea* (Valenciennes) collected in Lingao, Hainan province, China	2019	MV-4-11 (*biphenotypic B myelomonocytic leukemia*)<br>K562 (*erythroleukemic cell line*)	All human except B16F10 which is from mouse	3.94<br>6.05<br>3.10	47

						A673 (*rhabdomyoma cell line*)		8.67	
						U87 (*glioblastoma cell line*)		9.62	
						A549 (*lung*)		6.10	
						N87 (*gastric carcinoma*)		7.14	
						H1299 (*non-small cell lung carcinoma)*		11.32	
						HUCCT1 (*bile duct carcinoma*)		11.22	
						B16F10 (*highly metastatic mouse melanoma cell line*)			
						Karpass299		5.89	
	Ochrazepine B **(**Cpd**. 86)**					(*T cell lymphoma*)		9.91	
	Ochrazepine C **(**Cpd**. 87)**					U251(*glioblastoma*)		8.24	
						A673 (*rhabdomyoma cell line*)		9.04	
						U87 (*glioblastoma cell line*)		10.28	
								8.26	
	Ochrazepine D **(**Cpd**. 88)**					Hep3B (*liver cancer*)			
						U251 (*glioblastoma cell line*)		8.95	
	2-hydroxycircumdatin C (Cpd. **89**)								
	Aspyrone (Cpd. **90**)					U251 (*glioblastoma cell line*)		2.54	
						MV-4-11 (*biphenotypic B myelomonocytic leukemia*)		5.22	
						K562 (*erythroleukemic cell line*)		8.55	
						A673 (*rhabdomyoma cell line*)		4.57	
						N87 (*gastric carcinoma*)		5.83	
						H1299 (*non-small cell lung carcinoma)*		9.79	
						HUCCT1 (*bile duct carcinoma*)		5.89	
						B16F10 (*highly metastatic mouse melanoma cell line*)		2.57	
						Karpass299		5.48	
						(*T cell lymphoma*)		5.92	

						Hep3B (*liver cancer*)			
						A431 (*Epidermoid carcinoma cell line*)		6.32	
						143B (*bone osteosarcoma*)		5.79	
						MKN-45 (*gastric cancer*)		2.99	
						H1975 (*non-small cell lung carcinoma with L858R and T790M mutation*)		6.89	
						HL-60 (*promyelocytic leukemia*)		5.61	
						DU145 (*prostate cancer*)		9.31	
						SPC-A1 (*lung cancer over expressing maspin cell line*)			

Table S2. The IC_{50} of marine–derived terpenes and terpenoids with anticancer activity

S/N	Isolated Compounds	Class	Status (Known /Novel)	Source	Year	Cancer cell line	Source	IC_{50} (µM)	Ref
1.	Eutypellacytosporin A, $C_{40}H_{58}O_{11}$ (Cpd. **91**)	Meroterpenoids	All Novel	Arctic fungus Eutypella sp. D-1 from soil of London Island of ongsfjorden of Ny-Ålesund District in the Arctic.	2019	DU145 (*prostate*)<br>SW1990 (*pancreas*)<br>Huh7 (*liver)*<br>Panc-1 (*pancreas)*	All human	17.1<br>7.3<br>8.4<br>9.7	48
	Eutypellacytosporin B, $C_{40}H_{58}O_{11}$ (Cpd. **92**)					DU145 (*prostate*)<br>SW1990 (*pancreas*)<br>Huh7 (*liver)*<br>Panc-1 (*pancreas)*		11.0<br>4.9<br>4.9<br>7.9	
	Eutypellacytosporin C, $C_{40}H_{58}O_{11}$ (Cpd. **93**)					DU145 (*prostate*)<br>SW1990 (*pancreas*)<br>Huh7 (*liver)*<br>Panc-1 (*pancreas)*		13.5<br>9.6<br>11.2<br>10.2	
	Eutypellacytosporin D, $C_{40}H_{58}O_{11}$ (Cpd. **94**)					DU145 (*prostate*)<br>SW1990 (*pancreas*)<br>Huh7 (*liver)*<br>Panc-1 (*pancreas)*		13.4<br>8.2<br>9.6<br>7.5	

2.	Diorcinol (Cpd. **97**)	Indoloditerpenes	Both Known	Marine-derived fungus *Aspergillus versicolor* ZZ761	2019	U87MG (*glioma*)<br>U251(*glioma*)	Both human	4.4<br>6.2	49
	Versicolorin B (Cpd**. 101**)					U87MG (*glioma*)		11.3	
3.	Insulicolide A (Cpd. **114**)	Drimane sesquiterpenoid derivatives	Known	*Aspergillus. Flocculosus* obtained from marine sediment sample (Nha Trang Bay, South ChinanSea,Vietnam	2019	22Rv1 (*prostate*)	Human	3.0	50
						Neuro-2a (*neuron*)	Murine	4.9	
4.	19-methoxy-dictyoceratin-A (Cpd. **123**)	?	Novel	Marine sponge *Dactylospongia. elegans* was collected off Yongxing Island in the South China Sea	2019	Huh7 (*liver*)	All human	17.4	51
	Smenospongiarine (Cpd. **125**)		Known (Cpds. **125-128**)			DU145 (*prostate*)<br>SW1990 (*pancreas*)<br>Huh7 (*liver)*<br>Panc-1 (*pancreas)*		6.1<br>5.9<br>3.7<br>8.7	
	Smenospongorine (C. **126**)					DU145 (*prostate*)<br>SW1990 (*pancreas*)<br>Huh7 (*liver)*<br>Panc-1 (*pancreas)*		4.2<br>4.4<br>3.0<br>7.7	
	Smenospongimine (Cpd. **127**)					DU145 (*prostate*)<br>SW1990 (*pancreas*)<br>Huh7 (*liver)*<br>Panc-1 (*pancreas)*		3.5<br>4.2<br>2.3<br>5.8	
	Dictyoceratin-C (Cpd. **128**)					DU145 (*prostate*)<br>SW1990 (*pancreas*)<br>Huh7 (*liver)*<br>Panc-1 (*pancreas)*		8.3<br>7.9<br>6.9<br>9.2	
5.	Andrastone A (Cpd. **129**)	Meroterpenoids	Novel	Deep-Sea-Derived Fungus *Penicillium allii-sativi* from the deep-sea water of the western Pacific	2019	HepG2 (*liver*)	Human	7.8	52
6.	Sakurasosaponin (Cpd. **133**)	Triterpene saponin	Both known	Leaves of the mangrove *Aegiceras corniculatum* were collected from Bai Tu Long bay, Quang Ninh Province, Vietnam	2019	MCF-7 (*breast*)<br>A549 (*lung adenocarcinoma*)<br>B16F10 (*melanoma*)<br>HCT116 (*colon*)	All human	9.85<br>2.89<br>4.96<br>3.40	53
	Sakurasosaponin methyl ester (Cpd. **134)**					MCF-7 (*breast*)<br>A549 (*lung adenocarcinoma*)<br>B16F10 (*melanoma*)<br>HCT116 (*colon*)		9.86<br>5.45<br>18.27<br>2.21	
7.	Flaccidenol A (Cpd. **149**)	Capnosane (Cpd.**149**) cembranoids (Cpd **151**) -based diterpenoids	Novel (**149**, **151**)	From marine soft coral *Klyxum flaccidum*, collected off the coast of the island of Pratas	2019	A549 (*lung adenocarcinoma*)<br>DLD-1 (*colorectal adenocarcinoma*)	Human<br>Human<br>Mouse	9.7<br>6.0<br>7.2	54

	Flaccidodioxide (Cpd. **151**)<br>14-*O*acetylsarcophytol B (Cpd). **155**)		Known			P388D1 (*mouse lymphocytic leukaemia*)<br>P388D1(*mouse lymphocytic leukaemia*)<br>A549(*lung adenocarcinoma*)<br>DLD-1 (*colorectal adenocarcinoma*)<br>P388D1 (*mouse lymphocytic leukemia*)		19.6<br>10.8<br>11.7<br>8.9	
8.	Asterolaurin N (Cpd. **156)**<br>13-epi-9-desacetylxenicin (Cpd. **157**)<br>Xeniolide-B 9-acetate (Cpd. **158**)<br>Asterolaurin I (Cpd. **159**)	Diterpenes	Novel (Cpd. **156**)<br>Known (Cpds. **157-159**)	Taiwanese soft coral *Asterospicularia laurae*, southern coast of Taiwan	2019	Molt 4 (T *lymphoblast; acute lymphoblastic leukaemia*)<br>Molt 4 *(T lymphoblast; acute lymphoblastic leuakemia*)<br>K562 (*blood chronic myelogenous leukaemia*)<br>Sup-T1 (*T cell lymphoblastic lymphoma*)<br>U937 (*Caucasian histiocytic lymphoma*)<br>Molt 4 T *lymphoblast; acute lymphoblastic leuakemia*)<br>Molt 4 T *lymphoblast; acute lymphoblastic leuakemia*)	All human	19.41<br>1.30<br>1.19<br>3.17<br>2.45<br>15.13<br>18.75	55
9.	Cembranolide diterpene derivative 1 (Cpd. **160**)	Diterpenoid	Novel	Soft coral, *Lobophytum* sp. from the coast of Irabu Island, Okinawa, Japan	2019	HeLa (*cervix*)<br>A549 (*lung*)<br>B16-F10 (*melanoma*)<br>RAW 264.7 (*murine macrophage*)	Human (First 3) Murine	7.81<br>9.30<br>10.83<br>5.99	56
10.	Ambliol C (Cpd. **174**)	Furanoditerpenes	Known	Sponge *Spongia tubulifera* collected from the coast of the Mexican Caribbean	2019	MCF-7 (*breast*)<br>MiaPaca-2 (*pancreas*)	Both human	19.9<br>11.7	57
11.	Aphidicolin A (Gpd. **183**)	Diterpenoids	Novel	Deep-Sea-Derived Fungus *Botryotinia fuckeliana* MCCC 3A00494 western Pacific Ocean	2019	T24 (*Kidney*)<br>HL-60 (*Leukaemia*)	Both human	2.5<br>6.1	58

12.	Ilimaquinone (Cpd. **255**)	Sesquiterpene quinones and quinols	All known	Marine Sponge *Dactylospongia elegans* Sheraton Caverns, Kauai, Hawaiian	2019	U251MG (*glioblastoma*)	Both human	19.3	59
	5-*epi*-ilimaquinone (Cpd. **256**)					U251MG (*glioblastoma*)<br>Panc-1 (*pancreatic carcinoma*)		19.4<br>16.2	
	Smenospongine (Cpd. **257**)					U251MG(*glioblastoma*)		2.4	
	Smenospongorine (Cpd. **258**)					U251MG (*glioblastoma*)		19.4	
	Smenospongiarine (Cpd. **259**)					U251MG (*glioblastoma*)<br>Panc-1 (*pancreatic*		4.5<br>15.1	
	Smenospongidine (Cpd. **260**)					U251MG (*glioblastoma*)<br>Panc-1 (*pancreatic*		4.0<br>12.6	
	Dictyoceratin A (Cpd. **261**)					U251MG (*glioblastoma*)		2.8	
	Dictyoceratin B (Cpd. **262**)					U251MG (*glioblastoma*)		8.4	
	Dictyoceratin C (Cpd. **263**)					U251MG (*glioblastoma*)		4.1	
13.	Erythrolide D (Cpd. **268**)	Chlorinated briarane diterpenoids	All known	*Erythropodium caribaeorum* from soft coral collected from Caribbean sea in Colombia	2019	A549 (*lung*)	All human	2.58	60
	Erythrolide A (Cpd. **275**)					A549 (*lung*)<br>MCF-7 (*breast*)<br>PC3 (*prostate*)		18.41<br>6.77<br>2.45	
	Erythrolide B (Cpd. **276**)					MCF-7 (*breast*)<br>PC3 (*prostate*)		15.21<br>6.46	
14.	Henridilactone A (Cpd. **281**)	Nortriterpenoids (Cpd. **281**)<br>Lignan (Cpds. **287**, **289**)	Novel (Cpd. **281**)<br>Known (Cpds. **287**, **289**	*Schisandra sphenanthera* collected in KonTum province, Vietnam	2019	PC3 (*prostate*)<br>MCF-7 (*breast*)	Both human	3.21<br>13.30	61
	Schisandrathera C (Cpd. **287**)					PC3 (*prostate*)<br>MCF-7 (*breast*)		19.11<br>13.30	
	Schirubrisin B (Cpd. **289**)					PC3 (*prostate*)<br>MCF-7 (*breast*)		3.21<br>17.82	
15.	(+)-19-methylaminoavarone (Cpd. **290**)	Sesquiterpenoid quinones	Novel (Gpd. **290**)<br>Known (Cpds. **292-296**)	Marine sponge *Dysidea* sp from Xisha Islands, South China	2019	HCT-116 (*colon*)	All human	5.48	62
	(-)-20-methoxyneoavarone (Cpd. **291**)					A549 (*lung*)<br>HeLa (*cervix*)<br>HCT-116<br>Jurkat cell (*T-lymphocyte*)<br>K562 (*leukaemia*)<br>BEL-7402 (*hepatoma*)		4.61<br>0.93<br>1.31<br>2.29<br>3.07<br>4.02	
	(þ)-20-methoxyavarone (Cpd. **292**)					HCT-116 (*colon*)<br>Jurkat cell (*T-lymphocyte*)<br>K562 (*leukaemia*)		5.19<br>2.69<br>13.35	
	(-)-20-phenethylaminoavarone (Cpd. **294**)					A549 (*lung*)<br>HeLa (*cervix*)<br>HCT-116<br>Jurkat cell (*T-lymphocyte*)		7.66<br>4.88<br>3.52<br>12.58	

						K562 (*leukaemia*)<br>BEL-7402 (*hepatoma*)		9.81<br>9.40	
	(-)-20-methylaminoavarone (Cpd. **296**)					A549 (*lung*)<br>HeLa (*cervix*)<br>Jurkat cell (*T-lymphocyte*)		7.77<br>3.42<br>4.27	
16.	Penicindopene A (Cpd. **297**)	Andole diterpene	Novel	Deep-sea fungus *Penicillium* sp. YPCMAC1 from Yap Trench in West Pacific Ocean	2019	A549 (*lung*)	human	15.2	63
17.	14,15-dehydro-6-*epi*-ophiobolin K (Cpd. **305**)	Sesterterpenes	Novel (Cpd. **305-309**), Known (Cpd. **310-313**)	Fungus *Aspergillus flocculosus* 168ST-16.1 was isolated from the algae *Padina* sp., collected at a depth of 10 m in SonTra peninsular, Da Nang, Vietnam	2019	HCT-15(*colon*)<br>NUGC-3 (*stomach*)<br>NCI-H23 (*lung*)<br>ACHN (*renal*)<br>PC-3(*prostate*)<br>MDA-MB-231 (*breast*)	All human	0.21<br>0.19<br>0.18<br>0.24<br>0.24<br>0.14	64
	14,15-dehydroophiobolin K (Cpd. **306**)					HCT-15(*colon*)<br>NUGC-3 (*stomach*)<br>NCI-H23 (*lung*)<br>ACHN (*renal*)<br>PC-3(*prostate*)<br>MDA-MB-231 (*breast*)		0.44<br>0.50<br>0.61<br>0.53<br>0.47<br>0.63	
	14,15-dehydro-6-*epi*-ophiobolin G (Cpd. **307**)					HCT-15(*colon*)<br>NUGC-3 (*stomach*)<br>NCI-H23 (*lung*)<br>ACHN (*renal*)<br>PC-3(*prostate*)<br>MDA-MB-231 (*breast*)		0.96<br>0.88<br>1.40<br>1.14<br>1.00<br>1.05	
	14,15-dehydro-ophiobolin G (Cpd. **308**)					HCT-15(*colon*)<br>NUGC-3 (*stomach*)<br>NCI-H23 (*lung*)<br>ACHN (*renal*)<br>PC-3(*prostate*)<br>MDA-MB-231 (*breast*)		1.24<br>1.07<br>1.50<br>1.40<br>1.38<br>1.35	
	14,15-dehydro-(*Z*)-14-ophiobolin G (Cpd. **309**)					HCT-15(*colon*)<br>NUGC-3 (*stomach*)<br>NCI-H23 (*lung*)<br>ACHN (*renal*)<br>PC-3(*prostate*)<br>MDA-MB-231 (*breast*)		1.67<br>1.53<br>1.84<br>2.01<br>1.60<br>1.75	
	6-*epi*-ophiobolin C (Cpd. **310**)					HCT-15(*colon*)<br>NUGC-3 (*stomach*)<br>NCI-H23 (*lung*)<br>ACHN (*renal*)		0.24<br>0.22<br>0.24<br>0.43<br>0.27	

						PC-3(*prostate*)		0.19	
	Ophiobolin C (Cpd. **311)**					MDA-MB-231 (*breast*)<br>HCT-15(*colon*)<br>NUGC-3 (*stomach*)<br>NCI-H23 (*lung*)<br>ACHN (*renal*)<br>PC-3(*prostate*)		0.21<br>0.20<br>0.16<br>0.20<br>0.36<br>0.22	
	6-*epi*-ophiobolin N (Cpd. **312**)					MDA-MB-231 (*breast*)<br>HCT-15(*colon*)<br>NUGC-3 (*stomach*)<br>NCI-H23 (*lung*)<br>ACHN (*renal*)<br>PC-3(*prostate*)		0.30<br>0.22<br>0.22<br>0.23<br>0.20<br>0.21	
	Ophiobolin N (Cpd. **313**)					MDA-MB-231 (*breast*)<br>HCT-15(*colon*)<br>NUGC-3 (*stomach*)<br>NCI-H23 (*lung*)<br>ACHN (*renal*)<br>PC-3(*prostate*)<br>MDA-MB-231 (*breast*)		0.22<br>0.20<br>0.22<br>0.42<br>0.20<br>0.19	
18.	12-O-acetyl-nardosinan-6-en-1-one (Cpd. **314**)	Sesquiterpenes (Cpds. **314-320**)<br>Steroid (Cpd. **321**)	Novel (Cpds. **314-316**)<br>Known (Cpds **317-321**)	Soft coral Rhytisma. *fulvum fulvum* collected from the Red Sea Coast at Jeddah, Saudi Arabia	2019	NCI-H1299 (*lung*)<br>HepG-2 (*liver*)	All human	0.036<br>0.083	65
	6b-acetyl-1(10)-a-13-nornardosin-7-one (Cpd. **315**)					NCI-H1299 (*lung*)<br>HepG-2 (*liver*)		0.097<br>0.370	
	6,7-seco-13-nornardosinane (Cpd. **316**)					NCI-H1299 (*lung*)<br>HepG-2 (*liver*)		0.088<br>0.246	
	6a-acetyl-1(10)-a-13-nornardosin-7-one (Cpd. **317**)					NCI-H1299 (*lung*)<br>HepG-2 (*liver*)		0.097<br>0.370	
	12-Acetoxy-l(10)-aristolene (Cpd. **318**)					NCI-H1299 (*lung*)<br>HepG-2 (*liver*)		0.036<br>0.072	
	4-Acetoxy-2,8-neolemnadien-5-one (Cpd. **319**)					NCI-H1299 (*lung*)<br>HepG-2 (*liver*)		0.036<br>0.083	
	Cembranoid-diterpene Nephthenol (Cpd. **320**)					NCI-H1299 (*lung*)<br>MCF-7 (*breast*)		0.035<br>0.034	
	24-Methylcholesterol (Cpd. **321**)					MCF-7 (*breast*)		0.133	

Table S3. The IC_{50} of marine-derived amino acids, peptides, and polyketides with anticancer activity

S/N	Isolated Compounds	Class	Status (Known /Novel)	Source	Year	Cancer Cell line	Source	IC_{50} (µM)	Ref.
1.	$C_{31}H_{37}NO_7$ (Cpd. **323**)	Ansamycins (Divergolide) – Macrolides (polyketides)	All Novel	*Streptomyces* sp. KFD18 from Danzhou, Hainan province, China	2019	SGC-7901 (*gastric*)<br>K562 (*leukaemia*)<br>Hela (*cervix*)<br>A549 (*lung carcinoma*)	All Human	2.8<br>6.6<br>9.6<br>14.9	66
	$C_{31}H_{37}NO_8$ (Cpd. **324**)					SGC-7901 (*gastric*)<br>K562 (*leukaemia*)		9.8<br>9.0	
	$C_{31}H_{37}NO_7$ (Cpd. **325**)					SGC-7901 (*gastric*)<br>K562 (*leukaemia*)<br>Hela (*cervix*)		4.7<br>7.6<br>14.1	
	$C_{31}H_{37}NO_7$ (Cpd. **326**)					Hela (*cervix*)		16.3	
2.	Microcolin E (Cpd. **329**)	Lipopeptides (Cpds. **329-335** and **337**)<br>Microcolins deriv. (**342**, **343**)	Novel (Cpds. **329-335** and **337**)<br>Known (**338-341**)	The cyanobacteria (*Moorea producens*) samples were collected off Playa Kalki, Curacao	2019	H-460 (*lung*)	Human	1.0	67
	Microcolin F (Cpd. **330**)							0.037	
	Microcolin G (Cpd. **331**)							0.16	
	Microcolin H (Cpd. **332**)							0.047	
	Microcolin I (Cpd. **333**)							0.55	
	Microcolin J (Cpd. **334**)							0.069	
	Microcolin K (Cpd. **335**)							0.20	
	Microcolin M (Cpd. **337**)							0.51	
	Microcolin A (Cpd. **338**)							0.91	
	Microcolin B (Cpd.**3 39**)							0.65	
	Microcolin C (Cpd. **340**)							0.075	
	Microcolin D (Cpd. **341**)							2.80	
	3,4-dihydromicrocolins C (Cpd. **342**)							5.0	
	3,4-dihydromicrocolins D (Cpd. **343**)							2.0	
3.	Akazamicin (Cpd. **346**)	Aromatic polyketide	Novel	Nonomuraea sp. AKA32, marine-derived actinomycetes isolated from deep-sea water in Sagami Bay, Japan	2019	B16 (*melanoma*)	Murine	1.7	69
	Actinofuranone C (Cpd. **347**)		Known					1.2	
4.	Cytosaminomycin E (Cpd. **359**)	Pyrimidine nucleoside (Cpd. **361**) and derivatives (Cpds. **360-364**)	Novel	Marine-derived Streptomyces sp. SSA28 from Shengsi Archipelago (Zhejiang Province, China	2019	HCT-116 (*colon*)	Human	0.39	70
	40551-L (Cpd. **360**)		Known (**360-364**)					0.78	
	40551-K (Cpd. **361**)							6.45	
	40551-F (Cpd. **362**)							6.63	
	40551-D (Cpd. **363**)							2.14	
	40551-G (Cpd. **364**)							0.89	
5.	Fuscasins A (Cpd. **372**)	Cycloheptapeptides	Novel	Marine Sponge Phakellia fusca from from the South China Sea	2019	HepG2 (*liver*)	Human	4.6	71

6.	Julichrome Q6.6 (Cpd. **378**)	Julichrome derivative<br>Aromatic hydrocarbon	Both known	*Streptomyces* sp., NH1835 isolated from soil samples at the suburb of Wanning, Hainan province, China,	2019	HepG-2 (*hepatic*)<br>SMMC-7721 (liver)<br>MCF-7 (*breast*)<br>MDA-MB-231 (*breast*)	All Human	1.45<br>0.11<br>0.37<br>0.26	72
	Gliotoxin (Cpd. **382**)	Diketopiperazine<br>Amino acid				HepG-2 (*hepatic*)<br>SMMC-7721 (liver)<br>MCF-7 (*breast*)<br>MDA-MB-231 (*breast*)		13.18<br>1.89<br>0.99<br>0.92	
7.	Boshramycinones A (Cpd. **383**)	Anthracyclinones.<br>Polyketides	Novel	Marine-derived *Streptomyces* sp. Mei 16-1,2 from soil sample of Caeciliengroden salty marshland Wadden Sea at Jade Bay, North Sea coast of Germany)	2019	1218L(*bladder*)<br>T24 (*bladder*)<br>498N (*CNS*)<br>SF 268 (*CNS*)<br>HCT-116(*Colorectal*)<br>HT 29 (*Colorectal*)<br>251L (*Gastric*)<br>1121L (*Lung*)<br>289L (*Lung)*<br>526L (*Lung)*<br>529L (*Lung)*<br>629L (*Lung)*<br>H460 (*Lung)*<br>401NL (*Breast*)<br>MCF-7 (*Breast*)<br>276L (*melanoma*)<br>394NL (*melanoma*)<br>462NL (*melanoma*)<br>514L (*Melanoma*)<br>520L (*Melanoma*)<br>1619L (*Ovary*)<br>899L (*Ovary*)<br>OVCAR (*Ovary*)<br>Panc-1 (*Pancreas*)<br>22RV1 (*Prostate*)<br>DU145(*Prostate*)<br>LNCap (*Prostate*)<br>PC3M (*Prostate*)<br>1752L (*Pleuromesothelioma*)<br>393NL (*Kidney*)<br>486L (*Kidney*)<br>944L (*Kidney*)<br>1718L (*Kidney*)<br>1138L (*Uterus*)	All Human	1.11<br>1.14<br>0.86<br>0.95<br>1.47<br>0.95<br>1.31<br>1.09<br>2.76<br>2.56<br>1.18<br>1.64<br>1.35<br>1.34<br>1.26<br>1.46<br>0.90<br>1.29<br>0.97<br>3.29<br>1.20<br>4.87<br>0.66<br>1.06<br>0.51<br>1.66<br>1.04<br>0.99<br>6.75<br>1.06<br>12.18<br>1.63<br>2.62<br>0.93	74

	Boshramycinones B (Cpd. **384**)					MCF-7 (*breast*)<br>394NL (*Melanoma*)<br>22RV1 (*Prostate*)		14.30<br>17.27<br>12.91	
8.	16a-methylpregna-17a,19-dihydroxy-(9,11)-epoxy-4-ene-3,18-dione-20-acetoxy (Cpd. **392**)	Steroid	Known	Fungus *Penicillium citrinum* SCSIO 41017 associated with the sponge *Callyspongia* sp. from the sea area near Xuwen County, Guangdong Province, China	2019	MCF-7 (*breast*)	All human	1.3	75
	Penicitrinone A (Cpd. **396**)	Polyketide	Novel			SF-268 (*brain*)<br>MCF-7 (*breast*)<br>HepG-2 (*liver*)<br>A549 (*lung*)		13.7<br>17.2<br>13.5<br>18.0	
9.	Peniciversiol A (Cpd. **404**)	Polyketides	Novel (**404**, **407**)<br><br>Known (**408**-**419**)	Deep-Sea Derived Fungus *Penicillium chrysogenum* MCCC 3A00292	2019	BIU-87 (*liver*)	All human	10.21	76
	Penicilactone A (Cpd. **407**)					ECA109 (*oesophagus*)		12.41	
	Penicilactone B (Cpd. **408**)<br>3,3′-dihydroxy-5,5′-dimethyldiphenyl ether (Cpd. **411**)					ECA109 (*oesophagus*)		15.60	
	Violaceol-II (Cpd. **415**)					BIU-87(*liver*)		16.41	
	3,8-dihydroxy-4-(2,3-dihydroxy-1-hydroxymethylpropyl)-1-methoxyxanthone (Cpd. **416**)					ECA109 (*oesophagus*)<br>BEL-7402 (*hepatoma*)		8.95<br>15.94	
	Asperdemin (Cpd. **417**)					BEL-7402 (*hepatoma*)		12.75	
	Cyclopenol (Cpd. **418**)					BIU-87 (*liver*)<br>BEL-7402 (*hepatoma*)		8.34<br>7.81	
	Radiclonic acid (Cpd. **419**)					BIU-87 (*liver*)<br>ECA109 (*oesophagus*)<br>BEL-7402 (*hepatoma*)		12.47<br>7.70<br>13.75	
10.	Sekgranaticin (Cpd. **420**)	Polyketide	Novel (Cpd. **420**)<br>Known (Cpds. **421**-**423**)	*Streptomyces* sp. 166# isolated from clayey cold saline soil collected at County Naqu District, Tibet, China.	2019	MCF-7 (*breast*)<br>A549 (*lung*)<br>P6C (*colorectal*)<br>HCT-116 (*colon*)	All human	1.26<br>4.84<br>4.45<br>6.77	77
	Granaticins A (Cpd. **421**)					MCF-7 (*breast*)<br>A549 (*lung*)<br>P6C (*colorectal*)<br>HCT-116 (*colon*)		0.28<br>1.55<br>2.12<br>1.55	
	Granaticins B (Cpd. **422**)					MCF-7 (*breast*)<br>A549 (*lung*)<br>P6C (*colorectal*)<br>HCT-116 (*colon*)		0.37<br>3.57<br>0.28<br>0.01	
	Methyl granaticinate (Cpd. **423**)					MCF-7 (*breast*)		0.23	

						A549 (*lung*)<br>P6C (*colorectal*)<br>HCT-116 (*colon*)		1.39<br>0.88<br>0.02	
11.	Fusaperazine F (Cpd. **425**)	Diketopiperazine Cyclic dipeptides formed from N-terminal amino acid	Novel	Marine derived fungus *Penicillium crustosum* HDN153086 from Antarctic sediment from Prydz Bay	2019	K562 (*leukaemia*)	Human	12.7	78
12.	K41 A (Cpd. **430**)	Polyether polyketide	known	*Streptomyces species* Actinobacteria isolated from sediment sample obtained from Mersin Coastline (Turkey)	2019	CaCo-2 (*colorectal*)<br>PC-3 (*prostate*)	Both human	7.4<br>11.8	79
13.	Auxarthrol D (Cpd. **432**)	Anthraquinone derivatives (Polyketides)	Both novel	Fungal strain HDN16-802 isolated from the sediment sample of Zhangzi Island, from Dalian, Liaoning Province, China	2019	HL-60 (*leukaemia*)<br>HCT-116 (*colorectal*)<br>MDA-MB-231(*Breast*)<br>BEL-7402 (*hepatoma*)	All human	7.5<br>14.5<br>19.1<br>16.6	80
	Auxarthrol F (Cpd. **434**)					HL-60 (*leukaemia*)<br>Hela (*cervix*)<br>HCT-116 (*colorectal*)<br>MGC-803 (*gastric*)<br>HO8910 (*ovary*)<br>MDA-MB-231(*breast*)<br>SH-SY5Y (*Neuroblastoma*)<br>PC-3 (*prostate*)<br>K562 (*chronic myeloid leukemia*)		4.5<br>10.7<br>7.8<br>17.7<br>18.7<br>10.1<br>17.2<br>20.0<br>16.5	
14.	Jasplakinolide (Cpd **437**)<br>(+)-Jasplakinolide Z6 (Cpd. **440**)<br>(+)-Jasplakinolide Z4 (Cpd. **442**)<br>(+)-Jasplakinolide V (Cpd. **443**)	Cyclodepsipeptide	Novel (Cpd. **440**) Known (Cpds. **441**, **442**, **443**)	Marine sponge *Jaspis splendens* Samama, Panjang and Shoal islands of East Kalimantan (Indonesia)	2019	L5178Y (*melanoma*)	Mouse	< 0.01<br>3.2<br>< 0.01<br>< 0.01	82
15.	N-(3,7-dimethyl-2,6-octadienyl)-2-aza-2-deoxychaetoviridin A (Cpd. **445**)	Nitrogenated azaphilone polyketide	All novel	Deep-sea-derived fungus *Chaetomium globosum* MP4-S01-7 obtained from a water sample collected in the West Pacific Ocean	2020	MGC803 (*gastric*)<br>AGS (*gastric*)	Both human	0.78<br>0.12	83
	4′-epi-N-(3,7-dimethyl-2,6-octadienyl)-2-aza-2-deoxychaetoviridin A (Cpd. **446**)					MGC803<br>AGS		0.46<br>0.62	
	N-(3-methyl-2-butenyl)-2-aza-2-deoxychaetoviridin A (Cpd. **447**)					MGC803<br>AGS		2.7<br>6.5	

	4′-epi-N-(3-methyl-2-butenyl)-2-aza-2-deoxychaetoviridin A (Cpd. **448**)					MGC803<br>AGS		3.0<br>2.9	
	N-(3,7-dimethyl-2,6-octadienyl)-2-aza-2-deoxychaetoviridin E (Cpd. **449**)					MGC803<br>AGS		0.72<br>0.12	
	N-(3-methyl-2-butenyl)-2-aza-2-deoxychaetoviridin E (Cpd. **450**)					MGC803<br>AGS		6.8<br>2.0	
	4′,5′-dinor-5′-deoxy-N-(3,7-dimethyl-2,6-octadienyl)-2-aza-2-deoxychaetoviridin A (Cpd. **451**)					MGC803<br>AGS		2.2<br>1.2	
	4′,5′-dinor-5′-deoxy-N-(3-methyl-2-butenyl)-2-aza-2-deoxychaetoviridin A (Cpd. **452**)					MGC803		5.8	
16.	Epidithiodiketopiperazine · DC1149B (Cpd. **455**)	Piperazine	Novel	*Trichoderma lixii* isolated from an unidentifed marine sponge collected at Mentawai, Indonesia	2020	PANC-1 (*pancreas*)	Human	0.02	84
17.	Siphonellamides A (Cpd. **456**)	Acetylene amides	Both novel	Marine sponge, *Siphonochalina. siphonella* collected from the reefs southwest of Magawish Island, Hurghada, Egypt	2020	HeLa (*cervix*)<br>MCF-7 (*breast*)	Both human	9.4<br>18.0	85
	Siphonellamides B (Cpd. **457**)					HeLa (*cervix*)		17.4	
18.	Bulbimidazoles A (Cpd. **453**)	Alkanoyl imidazole	All novel	Coral sample *Tubastraea* sp., collected as fishery waste near the coast of Minami-Ise, Mie, Japan,	2020	P388 (*murine leukemia*)	Murine	5.0	86
	Bulbimidazoles B (Cpd **454**)							5.8	
	Bulbimidazoles C (Cpd. **465**)							7.0	
19.	Shellmycin A (Cpd. **466**)	Polyketides	All novel	*Streptomyces* sp. shell-016 isolated from the shell sediment of the Binzhou Shell Dike Island and Wetland National Nature Reserve, China	2020	A375 (*malignant melanoma*)<br>H1299 (*non-small cell lung cancer*)<br>HepG2 (*Hepatocellular carcinoma*)<br>HT29 (*colorectal adenocarcinoma*)<br>HCC1937 (*breast cancer*)	All human	0.69<br>1.32<br>0.89<br>0.85<br>2.62	87
	Shellmycin B (Cpd. **467**)					A375 (*malignant melanoma*)<br>H1299 (*non-small cell lung cancer*)<br>HepG2 (*Hepatocellular carcinoma*)<br>HT29 (*colorectal adenocarcinoma*)		0.95<br>2.62<br>1.35<br>1.12	

						HCC1937 (*breast cancer*)		3.11	
	Shellmycin C (Cpd. **468**)					A375 (*malignant melanoma*)		11.3	
						HepG2 (*Hepatocellular carcinoma*)		5.03	
						HT29 (*colorectal adenocarcinoma*)		4.33	
						HCC1937 (*breast cancer*)		12.6	
	Shellmycin D (Cpd. **469**)					A375 (*malignant melanoma*)		0.78	
						H1299 (*non-small cell lung cancer*)		2.15	
						HepG2 (*Hepatocellular carcinoma*)		1.11	
						HT29 (*colorectal adenocarcinoma*)		1.02	
						HCC1937 (*breast cancer*)		2.89	

Table S4. The IC_{50} of marine-derived lipids, sterols and steroids with anticancer activity

S/N	Isolated Compounds	Class	Status (Known /Novel)	Source	Year	Cancer cell line	Source	IC_{50} (µM)	Ref.
1.	16*β*-acetoxy-3*β*,7*β*,11*β*-trihydroxyergost-5,22-diene and named penicisteroid E (Cpd.**470**)	Steroids (**470**, **483**)<br>Steroids derivatives (**474-488**)	Novel (**470-474**)<br><br>Known (**475-488**)	Deep-sea-derived fungus *Penicillium granulatum* MCCC 3A00475<br>Antarctic Ocean.	2019	SHG-44 (*glioma*)<br>A549 (*non-small cell lung*)	All human	8.3<br>5.5	88
	16*β*-acetoxy-3*β*,5*α*,6*β*,7*β*-tetrahydroxyergost-22*E*-ene, and named penicisteroid G (Cpd. **473**)					SHG-44 (*glioma*)<br>Hep-G2 (*liver*)<br>A549 (*non-small cell lung*)<br>BIU-87 (*liver*)<br>BEL-7402 (*hepatoma)*<br>ECA-109 (*oesophagus*)<br>Hela-S3 (*cervix*)<br>Panc-1 (*pancreas*)		4.8<br>6.7<br>8.0<br>14.4<br>8.5<br>8.3<br>10.0<br>5.6	
	16*β*-acetoxy-3*β*,6*β*,7*β*-trihydroxyergost-22*E*-ene (Penicisteroid H) (Cpd. **474**)					Hep-G2 (*liver*)<br>A549 (*non-small cell lung*)<br>BIU-87 (*liver*)<br>ECA-109 (*oesophagus*)<br>Hela-S3 (*cervix*)		7.0<br>4.4<br>8.5<br>9.2<br>7.2	
	Penicisteroid A (Cpd. **475**)					SHG-44 (*glioma*)<br>Hep-G2 (*liver*)<br>A549 (*non-small cell lung*)<br>BIU-87 (*liver*)<br>BEL-7402 (*hepatoma*)<br>ECA-109 (*oesophagus*)<br>Hela-S3 (*cervix*)<br>Panc-1 (*pancreas*)		12.5<br>6.2<br>4.5<br>7.7<br>8.8<br>4.1<br>4.8<br>4.2	
	Penicisteroid C (Cpd. **476**)					SHG-44 (*glioma*)<br>HepG2 (*liver*)<br>ECA-109 (*oesophagus*)<br>Hela-S3 (*cervix*)<br>Panc-1 (*pancreas*)		7.8<br>4.4<br>6.6<br>9.9<br>7.0	
	Anicequol (Cpd. **477**)					A549 (*non-small cell lung*)			

	3*β*,5*α*,6*β*,22*E*)-6-methoxyergosta-7,22-diene-3,5-diol (Cpd. **481**)<br><br>5*α*,6*α*,8*α*,9*α*-diepoxy-(22*E*,24*R*)-ergoxt-22-ene-3*β*,7*β*-diol (Cpd. **482**)<br><br>Incisterol A2 (Cpd. **488**)					ECA-109 (*oesophagus*)<br>A549 (*non-small cell lung*)<br>BIU-87 (*liver*)<br>BEL-7402 (*bladder*)<br>Panc-1 (*pancreas*)<br>ECA-109 (*oesophagus*)<br>Hela-S3 (*cervix*)<br>HepG2 (*liver*)<br>BEL-7402 (*bladder*)<br>ECA-109 (*oesophagus*)<br>Hela-S3 (*cervix*)		5.9<br>15.6<br>7.0<br>8.7<br>8.0<br>6.3<br>7.1<br>9.8<br>16.6<br>7.9<br>7.5<br>8.2	
2.	2-hydroxyl-3-pyrenocine-thio propanoic acid (Cpd. **503**)<br><br>5,5-dichloro-1-(3,5-dimethoxyphenyl)-1,4-dihydroxypentan-2-one (Cpd. **506**)	Polysulfated steroids	Both novel	*Penicillum citreonigrum* XT20-134 (MCCC 3A00956) isolated from southeast Indian Ocean sediments	2019	Bel7402 (*Hepatoma tumor cell*)<br>HT1080 (*Fibrosarcoma tumor cell*)<br>Bel-7402 (*hepatoma*)<br>HT1080 (*Fibrosarcoma tumor cell*)	`All Human	7.63<br>13.14<br>10.22<br>16.53	89
3.	(24S)-ergosta-5,25-diene-3 < β > ,20,24,28-tetro l (Cpd. **526**)<br><br>1α,3β-dihydroxyergosta-5,24(28)-dien-7β-yl acetate (Cpd. **528**)	Oxygenated ergostane-type sterols	Novel	Isolated from the soft coral *Sinularia* sp. collected from the water area near the Xisha Islands, South China Sea	2019	MDA-MB-436 (*breast*)<br>H157 (*buccal mucosa*)<br>MDA-MB-436<br>Hep3B (*hepatic*)<br>HT-29 (*colorectal*)	Both human	17.5<br>10.17<br>18.14<br>19.30<br>10.38	90
4.	Eburicol (Cpd. **529**)	Steroid	known	Marine-derived fungi *Rhizopus* sp isolated from shellfish farming areas of the French Atlantic west coast	2019	MCF-7 (*breast*)<br>MDA-MB-231 (*breast*)	Both human	2.0<br>15.7	91
5.	Dokdolipid B (Cpd. **531**)	Hydroxylated rhamnolipids	Novel	Bacterial strain 179DD-027 *(Actinoalloteichus hymeniacidonis)* isolated from a sediment sample, collected off the coasts of Dokdo island, Republic of Korea	2019	HCT-15 (*colon*)<br>NUGC-3 (*stomach*)<br>NCI-H23 (*lung*)<br>ACHN (*renal*)<br>PC-3(*prostate*)	All human	16.7<br>19.3<br>13.7<br>14.1<br>18.2	92

molecules MDPI

Table S5. The IC_{50} of marine-derived ketones, quinines, quinolones and xanthones with anticancer activity

S/N	Isolated Compounds	Class	Status (Known /Novel)	Source	Year	Cancer cell line	Source	IC_{50} (µM)	Ref.
1.	3-methyl-antibiotic G-15F (Cpd. **535**)	Medermycin derivative	Known	*Streptomyces* sp. SS17A (from marine sediments of Shengsi Island (Zhejiangprovince, China)	2019	PC-3 (*prostate)*<br>HCT-116 (*colorectal*)	Human<br>Human	0.02<br>0.04	93
2.	Peniquinone A (Cpd. **541**)<br><br>Peniquinone B (Cpd. **543**)	1,4-benzoquinone (Aromatic)	Novel	Marine-derived fungus *Penicillium* sp. L129 from rhizosphere-soil of *Limonium sinense* (Girald) Kuntze collected in Yangkou Beach in Qingdao, China	2019	MCF-7 *(breast)*<br>U87 *(glioma)*<br>PC3 *(prostate)*<br>U87 *(glioma)*<br>PC3 *(prostate)*	All Human	12.39<br>9. 01<br>14.59<br>13.45<br>19.93	94
3.	Lithocarol A (Cpd. **558**)<br>Lithocarol B (Cpd. **559**)<br>Lithocarol D (Cpd. **561**)	Poly-ketal derivatives in tenellone family ketones	Novel	Marine-derived fungus *Phomopsis lithocarpus* FS508 Deep-sea sediment from Indian Ocean	2019	MCF-7 (*breast*)<br>A549 l*Lung*)<br>MCF-7m (*breast*)<br>HepG2 (*liver*)<br>A549 (*lung*)	Human	19.2<br>10.5<br>14.8<br>18.5<br>15.1	95
4.	Suberosanone B (Cpd. **567**	Bicyclic lactone	Known	Octocoral *Dendronephthya mucronata* collected near Phu Quoc Island, Kiengiang, Vietnam	2019	HeLa (*cervical adenocarcinoma*)	Human	14.45	96
5.	O-Demethylsterigmatocystin (Cpd. **586**)<br>Sterigmatocystin (Cpd. **587**)<br>Sterigmatin (Cpd. **588**)<br>AGI-B4 (Cpd. **589**)<br>Stephacidin A (Cpd. **594**)	Xanthone derivatives (Cpds. **586-589**)<br>Alkaloid (Cpd. **594**)	Known	Marine-derived fungus *Aspergillus. versicolor* (code F8.1.3a) isolated from the sponge *Agelas oroides,* which was collected from Aliağa-İzmir, Turkey co-cultured with *Bacillus. subtilis* lab strain 168 trpC2.	2019	L5178Y (*lymphoma*)	Mouse	5.8<br>2.2<br>2.3<br>2.0<br>16.7	97
6.	ULDF4 (kigamicin) (Cpd.**603**)<br>ULDF5 (staurosporine) (Cpd.**604**)	Xanthone<br>Indolocarbazole	Both known	*Strptomyces. bingchengensis* sediment samples collected from Folawiyo, Iddo in Lagos Lagoon, Nigeria	2019	HeLa (*cervical adenocarcinoma*)	Human	0.11<br>0.24	98

Table S6. The IC_{50} of other marine-derived compounds with anticancer activity

S/N	Isolated Compounds	Class	Status	Source	Year	Cancer Cell line	Source	IC_{50} (µM)	Ref.

			(Known /Novel)						
1.	7-oxo-holyrin A (Cpd.**605**)	Staurosporine derivatives Indololcarbazoles	Novel (Cpds **605-610**)<br><br>Known (Cpds. **611-619**)	*Streptomyces* sp. NB-A13 (from marine sediments of Ningbo City, Zhejiang province, China)	2019	PC-3 (*prostate*)<br>SW-620 (*colon*)	Both human	4.03<br>2.14	99
	4'-N-formyl-7-oxo-holyrin A (Cpd. **606**)					PC-3 (*prostate*)<br>SW-620 (*colon*)		2.05<br>0.74	
	3'-(hydroxyl(oxiran-2-yl)methoxy)-holyrine A (Cpd. **607**)					PC-3 (*prostate*)<br>SW-620 (*colon*)		2.45<br>2.00	
	3'-epi-5'-methoxy-K252d (Cpd. **608**)					PC-3 (*prostate*)<br>SW-620 (*colon*)		16.60<br>9.54	
	7-oxo-MLR-52 (Cpd. **609**)					PC-3 (*prostate*)<br>SW-620 (*colon*)		0.55<br>0.16	
	RK-1409 (Cpd. **611**)					SW-620 (*colon*)		0.010	
	Holyrine A (Cpd. **612**)					PC-3 (*prostate*)<br>SW-620 (*colon*)		2.06<br>0.76	
	3'-N-formyl-holyrin A (Cpd. **613**)					PC-3 (*prostate*)<br>SW-620 (*colon*)		2.50<br>0.73	
	MLR-52 (Cpd.**614**)					PC-3 (*prostate*)<br>SW-620 (*colon*)		0.56<br>0.18	
	k252d (Cpd. **615**)					PC-3 (*prostate*)<br>SW-620 (*colon*)		1.87<br>1.04	
	7-oxo-k252d (Cpd.**616**)					PC-3 (*prostate*)<br>SW-620 (*colon*)		2.27<br>0.81	
	3'-demethy-RK-1409 (Cpd. **617**)					PC-3 (*prostate*)<br>SW-620 (*colon*)		0.67<br>0.49	
	252a-Me (Cpd. **619**)					PC-3 (*prostate*)<br>SW-620 (*colon*)		0.77<br>0.02	
2.	Altercrasin B (Cpd. **621**)	Decalin derivatives	All novel	*Alternaria* sp. OUPS-117D-1 was isolated from the sea urchin *Anthocidaris Ccrassispana*, collected in Osaka bay, Japan	2019	P388 (l*ukaemia*)<br>HL-60 (l*ukaemia*)<br>L1210I (l*eukaemia*)	Murine<br>Human<br>Murine	20.0<br>12.1<br>8.0	100
	Altercrasin D (Cpd. **623**)					P388 (l*ukaemia*)<br>HL-60 (l*ukaemia*)<br>L1210I (l*eukaemia*)		9.7<br>6.1<br>8.4	
	Altercrasin E (Cpd. **624**)					P388 (l*ukaemia*)<br>HL-60 (l*ukaemia*)<br>L1210I (l*eukaemia*)		15.5<br>6.2<br>10.3	
3.	Nocarterphenyl A (Cpd. **625**)	Benzothiazole containing -(Cpd. **630** only) p-terphenyls and its derivatives (Cpds. **633** – **635**) aromatic hydrocarbon	Novel	Marine-Derived actinobacterial strain *Nocardiopsis* Sp. OUCMDZ-4936 from Dongzhaigang Mangrove Reserve, Hainan Province of China	2019	HL60 (*leukaemia*)<br>HCC1954 (*ductal carcinoma of the breast cells*)<br>MDA-MB-468 (*triple negative breast cancer*)	All Human	0.38<br>0.10<br><br>2.8	101

						H2228 (*lung*)		1.7	
						MV4-11 ((*TLT3-activated AML cells*)		4.0	
						K562 (*chronic myelogenous leukaemia*)		9.0	
						A549 (*lung*)		7.8	
						BT474 (*hormone sensitive breast cancer)*		6.0	
						A431(*epidermoid*)		4.6	
						MCF-7 (*breast*)		18.0	
						143B (*epidermoid carcinoma*)		5.5	
	5-methoxy-4,7-bis(4-methoxyphenyl)benzo[d]thiazol-6-ol (Cpd. **628**)		Known (Cpds **628-630**			K562 (*chronic myelogenous leukaemia*)		0.77	
						549 (*lung*)		5.1	
						MCF-7 (*breast*)		6.0	
						P6C (*CD44$^+$ colorectal stem cell line)*		9.4	
	2,4′,4″-trimethoxyp-terphenyl-3,6-dione (Cpd. **629**)					MV4-11 ((*TLT3-activated AML cells*)		0.16	
						A549 (*lung*)		0.48	
						HL60 (*leukaemia*)		0.17	
						MKN-45 (*gastric*)		0.49	
						N-87 (*gastric carcinoma*)		1.0	
						A673 (*rhabdomyoma cell line*)		0.76	
						K562 (*chronic myelogenous leukaemia*)		4.8	
						BT474 (*hormone sensitive breast cancer)*		3.6	
						H1229 (*non-small cell lung carcinoma*		0.72	
						HUCCT1(*bile duct carcinoma*)		0.20	
						B16F10 (*highly metastatic mouse melanoma cell line*)		0.76	
								1.1	

						MDA-MB-468 (*triple negative breast cancer*)<br>H-I975 (*lung*)<br>U251MG (*glioblastoma*)<br>HCC1954 (*ductal carcinoma of the breast cells*)<br>DU145 (*prostate*)<br>SPC-A1 (*lung cancer over expressing maspin cell line*)<br>HCT-116 (*colorectal*)<br>143B (*epidermoid carcinoma*)<br>H2228 (*lung*)<br>MDA-MB-231 (*breast*)		3.1<br>4.7<br>0.48<br>0.52<br>2.0<br>1.9<br>5.0<br>0.94<br>2.0	
	2,3,4′,4″-tetramethoxy-pterphenyl-6-ol (Cpd. **630**)					MV4-11 ((*TLT3-activated AML cells*)<br>MDA-MB-468 (*triple negative breast cancer*)<br>DU145 (*prostate*)<br>A673 (*rhabdomyoma cell line*)<br>K562 (*leukaemia*)<br>A549 (*lung*)<br>H1975 (*lung*)<br>HL-60 (*leukaemia*)<br>HCC1954 (*ductal carcinoma of the breast cells*)<br>MCF-7 (*breast*)<br>MKN-45 (*gastric*)<br>DU145 (*prostate*)<br>SPC-A1 (*lung cancer over expressing maspin cell line*)<br>143B (*lung cancer over expressing maspin cell line*)<br>H2228 (*lung*)		0.77<br>0.67<br>1.0<br>8.9<br>8.9<br>9.7<br>4.4<br>5.0<br>2.0<br>17<br>12<br>1.0<br>9.8<br>7.7	

						MDB-MB-231 (*breast*)		5.0<br>2.0	
4.	Terrosamycins A (Cpd. **631**)	Polycyclic polyether	Both novel	*Streptomyces* sp. RKND004 isolated from Prince Edward Island sediment	2019	HTB-26 (*breast adenocarcinoma*)<br>MCF-7 (*breast*)	Both human	10.1<br>6..0	102
	Terrosamycins B (Cpd. **632**)					HTB-26 (*breast adenocarcinoma*)<br>MCF-7 (*breast*)		6.0<br>3.9	
5.	Oxadiazine Nocuolin A (Cpd. **633**)	Azoles	known	Cyanobacterial strain LEGE 06071 from the LEGE CC culture (26) of CIIMAR, Porto, Portugal	2019	HCT116 (*colon*)<br>hTERT RPE-1 (immortalized epithelial cell line)	Both human	1.35<br>1.20	104
6.	Psolusoside L (Cpd. **638**)	Glycoside	Both novel	Sea cucumber *Psolus fabricii* (family Psolidae; order Dendrochirotid) were collected in the Sea of Okhotsk near Onekotan Island (Kurile Islands).	2019	Ehrlich ascites carcinoma (*liver*)<br>Neuro-2A (*neuroblastoma*)	All mouse	9.73<br>10.60	106
	Psolusoside N (Cpd. **640**)					Neuro-2A		13.52	
7.	Terphenyllin (Cpd. **646**)	*p*-terphenyls	Both known	Deep-sea-derived fungus Aspergillus candidus isolated from Atlantic Ocean	2019	HeLa (*cervix*)<br>ECA-109 (*oesophagus*)<br>Bel-7402 (*hepatoma*)<br>Panc-1 (*pancreas*)	All human	9.2<br>7.0<br>9.4<br>7.8	107
	Prenylterphenyllin (Cpd. **647**)					HeLa (*cervix*)<br>ECA-109 (*oesophagus*)<br>Bel-7402 (*hepatoma*)<br>Panc-1 (*pancreas*)		5.6<br>5.5<br>8.5<br>7.7	
8.	Oxalicumone A (Cpd. **656**)	Dihydrothiophene	Known	Fungus *Aspergillus* sp. LS34 isolated from sponge *Haliclona* sp. collected at Lingshui, Hainan Province, China	2019	CCRF-CEM (*human acute lymphoblastic leukemia T lymphocyte)*<br>K562 (*chronic myeloid leukemia*)	Both human	1.22<br>10.58	108
9.	Holothurin A_2 (Cpd. **659**)	Triterpene Glycoside	All known	Sea cucumber *Holothuria (Halodeima) edulis Lesson*, 1830 collected from Cu Lao Cham Island, Quangnam, Vietnam	2019	LNCaP (*prostate*)<br>HepG2 (*liver*)<br>KB (*epidermoid carcinoma*)<br>MCF-7 (*breast*)<br>SK-MEL-2 (*skin*)	All human	0.96<br>0.76<br>0.75<br>0.81<br>0.84	109
	Holothurin A (Cpd. **663**)					LNCaP (*prostate*)<br>HepG2 (*liver*)<br>KB (*epidermoid carcinoma*)<br>MCF-7 (*breast*)		1.30<br>2.03<br>1.79<br>2.29	

						SK-MEL-2 (*skin*)		2.49	
	Holothurin B (Cpd. **664**)					LNCaP (*prostate*)<br>HepG2 (*liver*)<br>KB (*epidermoid carcinoma*)<br>MCF-7 (*breast*)<br>SK-MEL-2 (*skin*)		2.74<br>2.63<br>2.75<br>3.35<br>3.66	
10.	Dragocin A (Cpd. **666**)	Ribofuranose	Novel	Marine *cyanobacterium* collected in Boca del Drago, Panama	2019	H-460 (*lung*)	Human	7.6	110
11.	Terrein (Cpd. **670**)	Not available	Known	*Aspergillus terreus* Strain S020 isolated from deep-sea sediment marine sponge collected from the Red Sea at Obhur, Jeddah, Saudi Arabia	2019	HCT-116 (*colorectal*)	Human	12.13	111
12.	Prenylterphenyllin F (Cpd. **671**)	Prenylterphenyllins	All novel	Endophytic fungus, *Aspergillus candidus* LDJ-5 isolated from the root of *Rhizophora apiculata* Blume collected from Sanya Bailu Park of Hainan Province, China	2020	MGC-803 (*Gastric*)<br>HCT-116 (*colorectal*)<br>BEL7402 (*hepatoma*)<br>A549 (*Lung*)<br>SH-SY5Y (*Neuroblast*)<br>HeLa (*cervix*)<br>HL-60 (*lukaemia*)	All human	11.0<br>9.3<br>12.4<br>10.2<br>10.4<br>8.3<br>7.1	112
	Prenylterphenyllin G (Cpd.**672**)					MGC-803 (*Gastric)*<br>HCT-116 (*colorectal*)<br>BEL7402 (*hepatoma*)<br>A549 (*Lung*)<br>SH-SY5Y (*Neuroblast*)<br>HeLa (*cervix*)		12.5<br>16.9<br>12.6<br>16.3<br>12.4<br>11.5	
	Prenylterphenyllin H (Cpd. **673**)					MGC-803 (*Gastric)*<br>HCT-116 (*colorectal*)<br>A549 (*Lung*)<br>SH-SY5Y (*Neuroblast*)<br>HeLa (*cervix*)<br>U87		0.7<br>0.5<br>0.4<br>0.6<br>2.0<br>13.8	
	Prenylterphenyllins J (Cpd. **675**)					MGC-803 (*Gastric)*<br>HCT-116 (*colorectal*)<br>A549 (*Lung*)<br>SH-SY5Y (*Neuroblast*)		8.1<br>6.2<br>7.6<br>15.6	

	Prenylcandidusin G (Cpd.**679**)					HeLa (*cervix*)<br>K562 (*leukemic*)<br>MGC-803 (*Gastric*)<br>HCT-116 (*colorectal*)<br>BEL7402 (*hepatoma*)<br>A549 (*lung*)<br>SH-SY5Y (*Neuroblast*)<br>HeLa (*cervix*)<br>HL-60 (*lukaemia*)		8.5<br>15.9<br>1.4<br>0.9<br>16.0<br>2.8<br>2.2<br>10.1<br>3.4	
13.	Monarubin B (Cpd. **683**)<br>Lunatinin (Cpd. **686**)	Isocoumarin	Novel<br>Known	Marine fungus *Monascus ruber* BB5isolated from *Meretrix meretrix* collected from Hailing Island, Yangjiang, China	2020	HepG-2 (*liver*)<br>QGY77011(*liver*)<br>HepG-2 (*liver*)<br>QGY77011(*liver*)	Both human	1.72<br>0.71<br>9.60<br>7.12	113
14.	Meleagrin (Cpd. **693**)	Unknown	Novel	Fungal strain *Emericella dentata* Nq45 was isolated from sediment sample obtained from the sea water of Nabq bay, Red Sea, Egypt	2020	KB-3-1 (*cervix carcinoma*)<br>KB-V1 (*multidrug resistant sub-clone of KB-3-1*)	Both human	3.07<br>6.07	114
15.	5,5′-oxybis(1-methoxy-3-methylbenzene) (Cpd.**711**)<br>4′-[(2-Hydroxy-1,3-propanedlyl)bis[oxy-4,1-phenylene(1-methylethylidene)]]bisphenol (Cpd. **722**)	Aromatic compounds	Both Known	Marine fungus *Trichoderma erinaceum* F1-1 isolated from inner tissue of the sea star *Acanthaster planci* collected from Hainan Sanya National Coral Reef Reserve, China		MDA-MB-435 (*melanocyte*)<br>A549 (*lung*)	Both human	12.5<br>18.4	115
16.	Pyrenosetins A (Cpd. **725**)<br>Pyrenosetins B (Cpd. **726**)	Decalinoylspirotetramic acid derivatives	Both novel	Fungal strain isolated from brown alga *Fucus vesiculosus* specimens collected in Falckenstein Beach Kiel Fjord, Baltic Sea, Germany	2020	A-375 (*malignant melanoma*)	Human	2.8<br>6.3	116
17.	Donghaecyclinones C (Cpd. **731**)	Angucyclinone	Novel	*Streptomyces* sp. strain SUD119, which was isolated from a marine sediment sample collected from a volcanic island in Korea	2020	HCT116 (*colorectal*)<br>MDA-MB231 (*breast*)<br>SNU638 (*gastric*)<br>A549 (*lung*)<br>SK-HEP1(*liver*)	All human	8.0<br>6.7<br>9.5<br>9.6<br>6.0	117
